# Ultra-fast Proton Conduction and Photocatalytic Water Splitting in a Pillared Metal–Organic Framework

**DOI:** 10.1021/jacs.3c03943

**Published:** 2023-08-22

**Authors:** Jin Chen, Bing An, Yinlin Chen, Xue Han, Qingqing Mei, Meng He, Yongqiang Cheng, Inigo J. Vitorica-Yrezabal, Louise S. Natrajan, Daniel Lee, Anibal J. Ramirez-Cuesta, Sihai Yang, Martin Schröder

**Affiliations:** † Department of Chemistry, The University of Manchester, Manchester M13 9PL, U.K.; ‡ Neutron Scattering Division, Neutron Sciences Directorate, Oak Ridge National Laboratory, Oak Ridge, Tennessee 37831, United States; § Department of Chemical Engineering and Analytical Science, University of Manchester, Manchester M13 9PL, U.K.; ∥ College of Chemistry and Molecular Engineering, Beijing National Laboratory for Molecular Sciences, Peking University, Beijing 100871, China

## Abstract

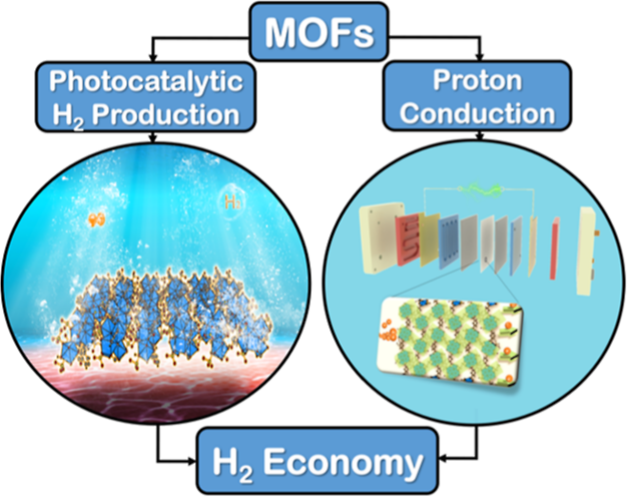

Proton-exchange membrane fuel cells enable the portable utilization of hydrogen (H_2_) as an energy resource. Current electrolytic materials have limitation, and there is an urgent need to develop new materials showing especially high proton conductivity. Here, we report the ultra-fast proton conduction in a novel metal–organic framework, MFM-808, which adopts an unprecedented topology and a unique structure consisting of two-dimensional layers of {Zr_6_}-clusters. By replacing the bridging formate with sulfate ligands within {Zr_6_}-layers, the modified MFM-808-SO_4_ exhibits an exceptional proton conductivity of 0.21 S·cm^–1^ at 85 °C and 99% relative humidity. Modeling by molecular dynamics confirms that proton transfer is promoted by an efficient two-dimensional conducting network assembled by sulfate–{Zr_6_}-layers. MFM-808-SO_4_ also possesses excellent photocatalytic activity for water splitting to produce H_2_, paving a new pathway to achieve a renewable hydrogen-energy cycle.

## Introduction

The achievement of global net zero targets relies on the replacement of fossil fuels with clean energy sources.^1^ Hydrogen (H_2_) can be produced from water and its combustion yields water as the exclusive product; this cycle is, therefore, regarded as a potential, widely accessible, and renewable energy source with zero emission at point of use. In addition, H_2_ possesses a high gravimetric energy density of 141.8 × 10^6^ kJ·kg^–1^, greater than most fuels such as gasoline (44 × 10^6^ kJ·kg^–1^) at room temperature.^2^ Light-driven water splitting is the most sustainable and targeted approach to the production of H_2_ and is continuing to attract much interest as part of the roadmap to the Hydrogen Economy.^3^ A parallel challenge is the release of energy that is stored within H_2_ molecules, and proton exchange membrane fuel cells (H_2_-PEMFCs) can convert this chemical energy to electricity with high efficiency.^4^ Within a PEMFC, the proton exchange membrane (PEM) is a crucial component to enable high performance and efficiency for energy conversion. Nafion, a sulfonate tetrafluoroethylene-based polymer, has been used for decades as a commercial PEM. However, despite its excellent proton conductivity [0.05 S·cm^–1^ at 98% relative humidity (RH) and 25 °C],^5^ Nafion has inherent drawbacks. The requirement of adequate hydration limits the operating efficiency and temperature (<80 °C), and its amorphous structure severely hinders the understanding of the mechanisms of proton-conduction and of associated structure–property relationships, thus restricting the design and development of improved PEM materials.^6^

Metal–organic framework (MOF) materials afford a versatile platform for tuning both proton transport and catalysis.^7,8^ To date, many crystalline MOFs with super-protonic conductivity (≥10^–4^ S·cm^–1^) have been reported,^9^ but examples of MOFs with ultra-high super-protonic conductivities (≥0.1 S·cm^–1^) remain extremely rare.^10^ Our approach to the design of a MOF showing high proton conductivity is to incorporate sufficient protonic sources and sites, coupled to efficient proton-hopping pathways *via* accessible hydrogen-bonding networks that enable protons to flow through the system rapidly. Currently, proton-conductive MOFs are based upon one- (1D) or three-dimensional (3D) networks, with the former dominating the field.^11,12^ MOFs with layered two-dimensional (2D) structures have also been studied for proton conduction. However, the 2D layers are assembled typically by metal nodes connected by extended and proton-insulating organic linkers, thus preventing protons from transferring freely throughout the entire layer.^13^ Thus, proton conduction in layered MOFs has been enabled by the diffusion of guest molecules residing in the interlayer space to give a *pseudo*-2D proton-conducting network.^14^ Conventional 2D-structured materials, such as graphene, graphene oxide, and boron nitride, show high proton conductivities in the range 10^–3^ to 10^–1^ S·cm^–1^, demonstrating the promise of 2D networks and topologies for rapid proton conduction.^15−17^

Here, we report a novel Zr-based MOF, MFM-808 (MFM = Manchester Framework Material), which shows an unprecedented ***mfm*** topology with a unique layered structure comprising {Zr_6_}-clusters bridged by formate ligands. Upon a single-crystal-to-single-crystal transformation, MFM-808 can be sulfated to yield MFM-808-SO_4_, where the formate bridges are fully replaced by sulfate ligands. The bridging sulfates within the layers of {Zr_6_} clusters serve as strong Brønsted acid centers, but also contribute to constructing efficient hydrogen-bonding networks due to multiple hydrogen donor/acceptor sites. This ordered sulfate-{Zr_6_}-cluster-layered structure constitutes a 2D proton-conducting network, as revealed by molecular dynamics (MD) simulation, and exhibits an exceptional proton conductivity of 0.21 S·cm^–1^ at 85 °C and 99% RH, outperforming Nafion and most state-of-the-art MOFs.^10^ More interestingly, MFM-808-SO_4_ can also function as an efficient photocatalyst to drive water splitting to produce H_2_ with an average rate for the hydrogen evolution reaction (HER) of 670 μmol·g^–1^·h^–1^. Therefore, MFM-808-SO_4_ offers the potential of combining cycles of proton transport and H_2_ production within the same platform.

## Results and Discussion

### Synthesis and Structure

**Figure 1:**
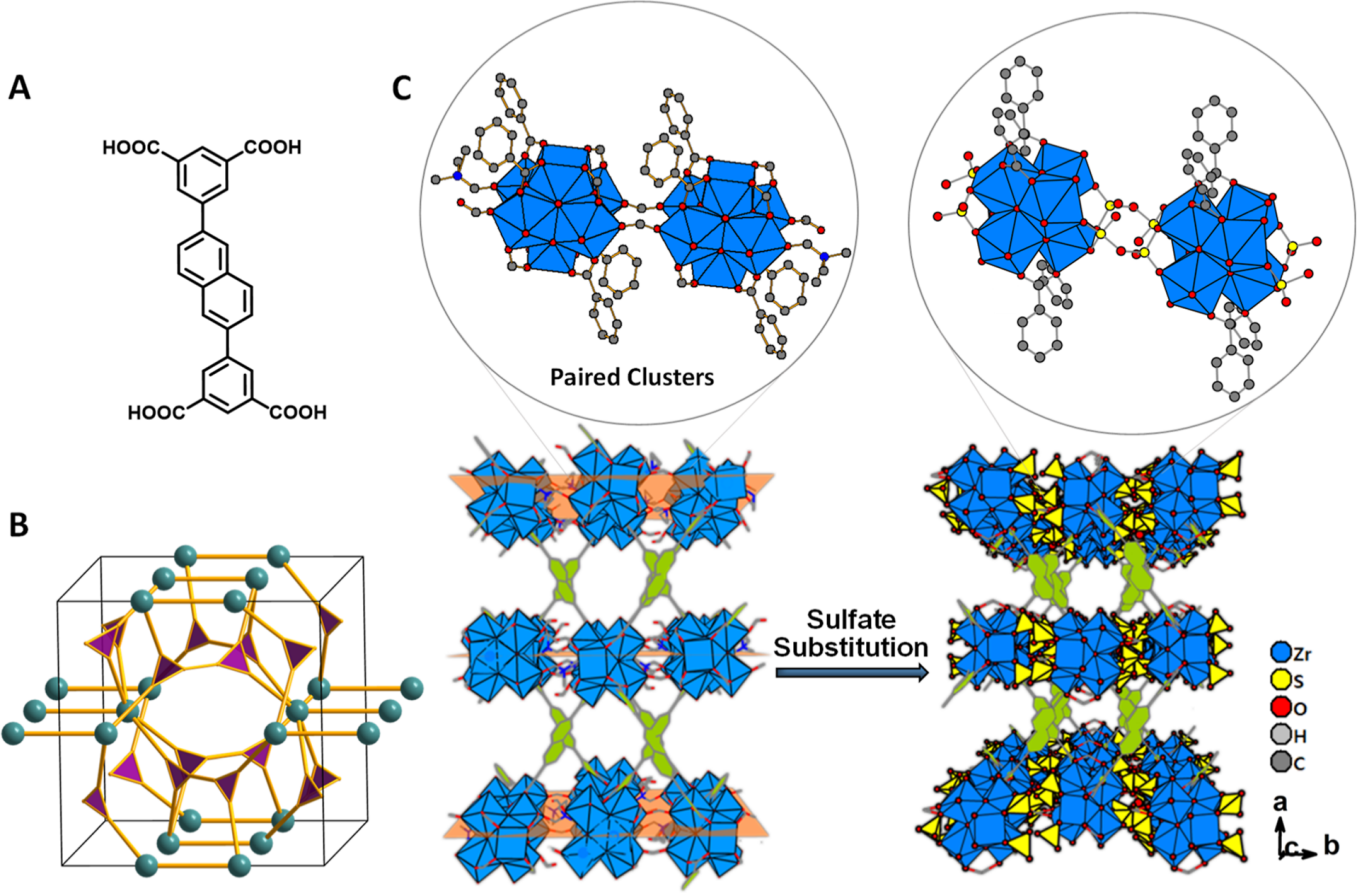
Structure of the organic linker and MFM-808 and MFM-808-SO_4_. (A) Structure of linker H_4_L. (B) View of the new ***mfm*** topology in MFM-808 and MFM-808-SO_4_. (C) Illustration of the {Zr_6_}_2_ pair, {Zr_6_}-cluster layer, and 3D structure viewed along the *c*-axis of MFM-808 and MFM-808-SO_4_. Zr, blue; S, yellow; O, red; H, light gray; C, dark gray; sulfate, yellow tetrahedral; and free solvent molecules in the pore are omitted for clarity.

MFM-808 was prepared by the hydrothermal reaction of a mixture of ZrOCl_2_, H_4_L [5,5′-(naphthalene-2,6-diyl)diisophthalic acid; Figures 1A and S1; Scheme S1], *N*,*N*′-dimethylformamide (DMF), and formic acid at 130 °C for 2 days (Scheme S2). Both single crystals and microcrystalline powder can be isolated in pure phase (Figures S2–S6), and the crystal structure was determined by single crystal X-ray diffraction (SC-XRD). MFM-808, [Zr_6_O_4_(OH)_6.5_(H_2_O)_2_(DMF)(HCOO)_5.5_L], crystallizes in the monoclinic *I*2/*a* space group, with the linker (L^4–^) of low C_2*h*_ symmetry being translated into the low symmetry of the resultant framework.^18^ The octahedral [Zr_6_O_4_(OH)_4_] cluster is formed *via* assembly of six Zr^4+^ ions with combinations of μ_3_-O and μ_3_-OH bridges. However, instead of forming one of the commonly observed 6-/8-/10-/12-fold connectivities, the [Zr_6_O_4_(OH)_4_] cluster in MFM-808 shows a rare five-fold connectivity with each {Zr_6_} cluster connected to a neighboring {Zr_6_} cluster *via* bridging formate and to four different L^4–^ ligands (Figure 1B). This yields a (3-c)_2_(5-c) binodal net with the new topology **mfm** (Figure 1B, point symbol for the net: {4·8^2^}_2_{4^2^·8^4^·10^4^}).^19,20^ The structure of MFM-808 can also be viewed as two {Zr_6_} clusters bridged by formate ligands to form pairs of {Zr_6_}_2_ moieties assembled into a dense layer of {Zr_6_}_2_ pairs in the *bc*-plane (Figures 1C, S7, and S8). These layers are pillared further by L^4–^ ligands, where each {Zr_6_} cluster is connected by four L^4–^ ligands along the *a*-axis to afford a framework structure and creating porosity between layers (Figures 1 and S9). The pore size of MFM-808 measured crystallographically is 6–8 Å (Figure S10), consistent with that derived from N_2_ sorption isotherms (Figure S11). Overall, the layered structure of MFM-808 is distinct to other reported Zr-based MOFs in which {Zr_6_} clusters are usually linked by extended organic ligands to afford 3D structures with well-defined cages and pores.^19^

Low connectivity often promotes flexibility in MOFs despite the rigidity of organic ligands applied.^21^ MFM-808 can be activated under dynamic vacuum at 130 °C to give the desolvated material, which was reacted with chlorosulfonic acid in CH_2_Cl_2_ (Scheme S2, Figures S2–S6) to give MFM-808-SO_4_. SC-XRD confirms MFM-808-SO_4_, [Zr_6_O_4_(OH)_4_(H_2_O)_8.4_(SO_4_)_2.5_L·(HSO_4_)_3_·(H_2_O)_8.3_], to have the same framework topology as MFM-808 but with the bridging formates between {Zr_6_} clusters replaced by sulfates to afford {Zr_6_}–sulfate–{Zr_6_} bridges (Table S1, Figures S7 and S8). Elemental analysis confirms a sulfur content of 9.4% for MFM-808-SO_4_, in good agreement with the crystallographic result (8.9%). Fourier transform infrared spectroscopy shows the presence of sulfate in MFM-808-SO_4_ (Figure S12) with the symmetric and asymmetric stretching modes of sulfate observed between 1000 and 1200 cm^–1^.^22^ Thermogravimetric analysis shows an additional weight loss in the range of 500–700 °C for MFM-808-SO_4_ compared with MFM-808, corresponding to the decomposition of sulfate (Figures S13 and S14). Solid-state magic angle spinning NMR spectra confirmed the presence of protonated sulfate in MFM-808-SO_4_ (Figure S15), consistent with the analysis of potentiometric acid–base titration (Figure S16). The protonated sulfate species can act as effective sources of protons to benefit and enhance proton conduction in porous MOFs.^23,24^ N_2_ sorption isotherms reveal a notable decrease in the Brunauer–Emmett–Teller surface area on going from MFM-808 (502 m^2^·g^–1^) to MFM-808-SO_4_ (47 m^2^·g^–1^), and the quasi-linear profile of the isotherm of MFM-808-SO_4_ suggests that the pores in MFM-808-SO_4_ are occupied by additional sulfate species, with a 1:3 molar ratio of {Zr_6_} clusters to adsorbed sulfate species being observed (Figure S17).^25,26^ The hydrophilic sulfate groups in MFM-808-SO_4_ also lead to an improved ability to absorb water, thus facilitating the construction of hydrogen-bonding network and thereby enhancing further the proton conduction within the system (Figure S18).^5^

### Proton Conductivity

**Figure 2:**
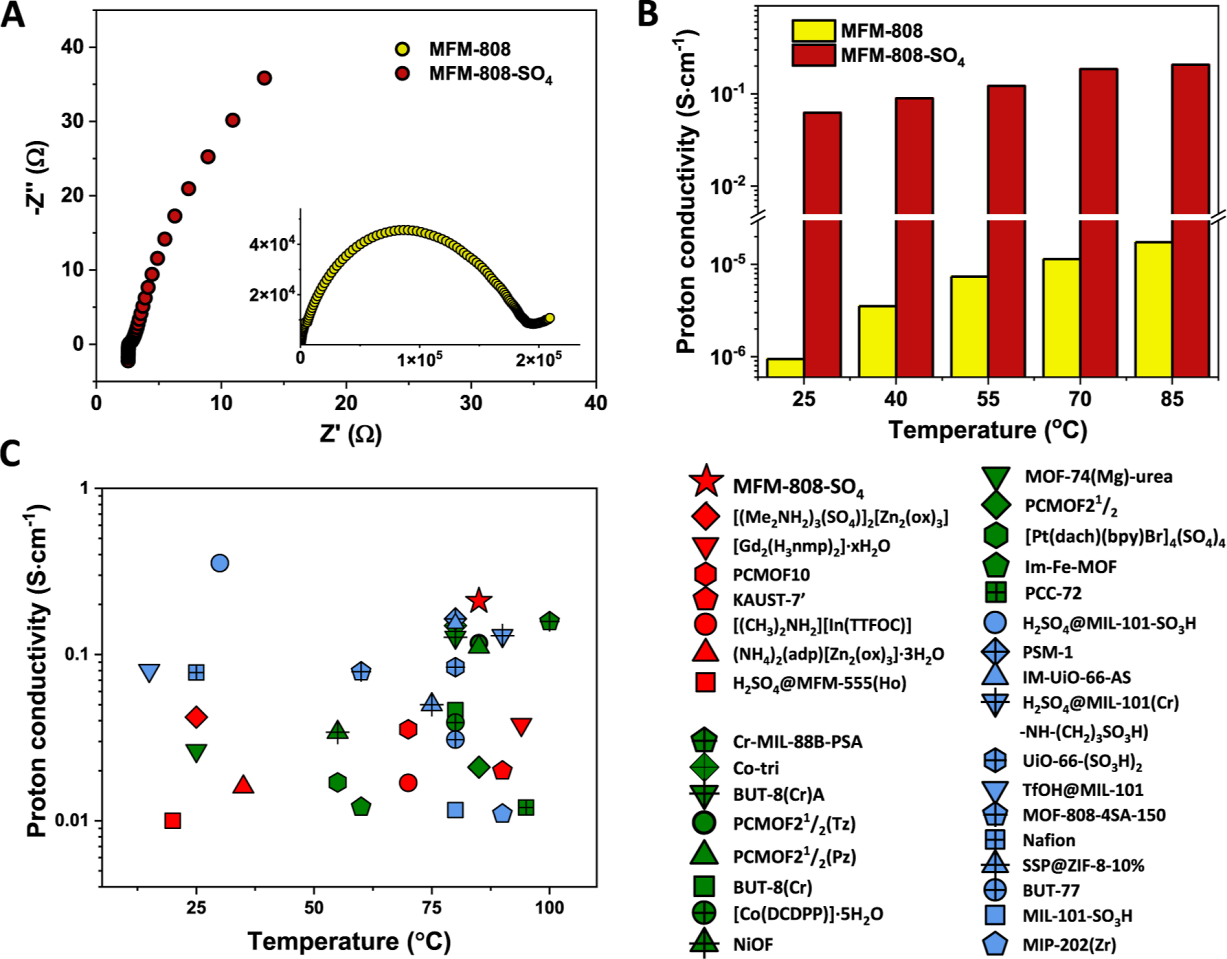
Proton conductivity of MFM-808 and MFM-808-SO_4_. (A) Nyquist plots of MFM-808 and MFM-808-SO_4_ measured at room temperature and 99% RH. (B) Proton conductivity of MFM-808 and MFM-808-SO_4_ as a function of temperature under 99% RH. (C) Comparison of excellent proton conductivity (>10^–2^ S·cm^–1^) of reported MOF materials with 1D (green), 2D (red), and 3D (blue) networks. Full data are given in Table S2.

The proton conductivity (σ) of MFM-808 and MFM-808-SO_4_ was evaluated using alternating current (AC) impedance spectroscopy. MFM-808 showed a maximum value for σ of 1.74 × 10^–5^ S·cm^–1^ at 85 °C and 99% RH (Figures S19 and S20), which is comparable to many pristine MOFs.^27^ Significantly, an exceptional value for σ of 0.21 S·cm^–1^ (85 °C, 99% RH) was observed for MFM-808-SO_4_ (Figures S21 and S22), an increase of >10,000 times compared with MFM-808, confirming the positive impact of sulfation of the {Zr_6_} cluster layers. To date, only a handful of MOFs have been reported to possess σ > 0.1 S·cm^–1^ (Figure 2, Table S2), and MFM-808-SO_4_ outperforms most of them as well as the benchmark, Nafion.^4,10^ Only one MOF, H_2_SO_4_@MIL-101-SO_3_H, shows a higher proton conductivity than MFM-808-SO_4_.^5^ However, it is challenging to visualize the proton-conducting pathway in this system due to the large cages (up to 4 nm) that are occupied by disordered H_2_SO_4_ molecules, and its performance was limited to below 70 °C. It is also worth noting that MFM-808-SO_4_ remains moderately proton-conductive at relatively low humidity at 25 °C (Figure S23). For example, MFM-808-SO_4_ exhibited a value for σ of 2.9 × 10^–4^ S·cm^–1^ at 60% RH, 25 °C, thus maintaining a super-protonic level (>10^–4^ S·cm^–1^).^28^ Such a good performance over a wide range of humidity is important for practical applications.^4^

The activation energy (*E*_a_) for proton transfer was obtained to define the conduction mechanism in these materials. The value of *E*_a_ for MFM-808 was calculated to be 0.46 eV (Figure S20), suggesting a vehicle mechanism (*E*_a_ > 0.4 eV) where protons are proposed to move *via* diffusion of protonated guest molecules that acted as proton carriers, *e.g.*, water.^6^ In comparison, MFM-808-SO_4_ shows a value for *E*_a_ of 0.22 eV (Figure S22), which is notably lower than that for H_2_SO_4_@MIL-101-SO_3_H (0.39 eV).^5^ This indicates a Grötthuss mechanism for MFM-808-SO_4_ involving proton conduction *via* hopping through a well-connected hydrogen-bonding network.^14^ Such a mode of conduction does not require carrier molecules and results in more rapid proton transfer in MFM-808-SO_4_ compared with MFM-808.^29−31^

### Proton-Conducting Mechanism

**Figure 3:**
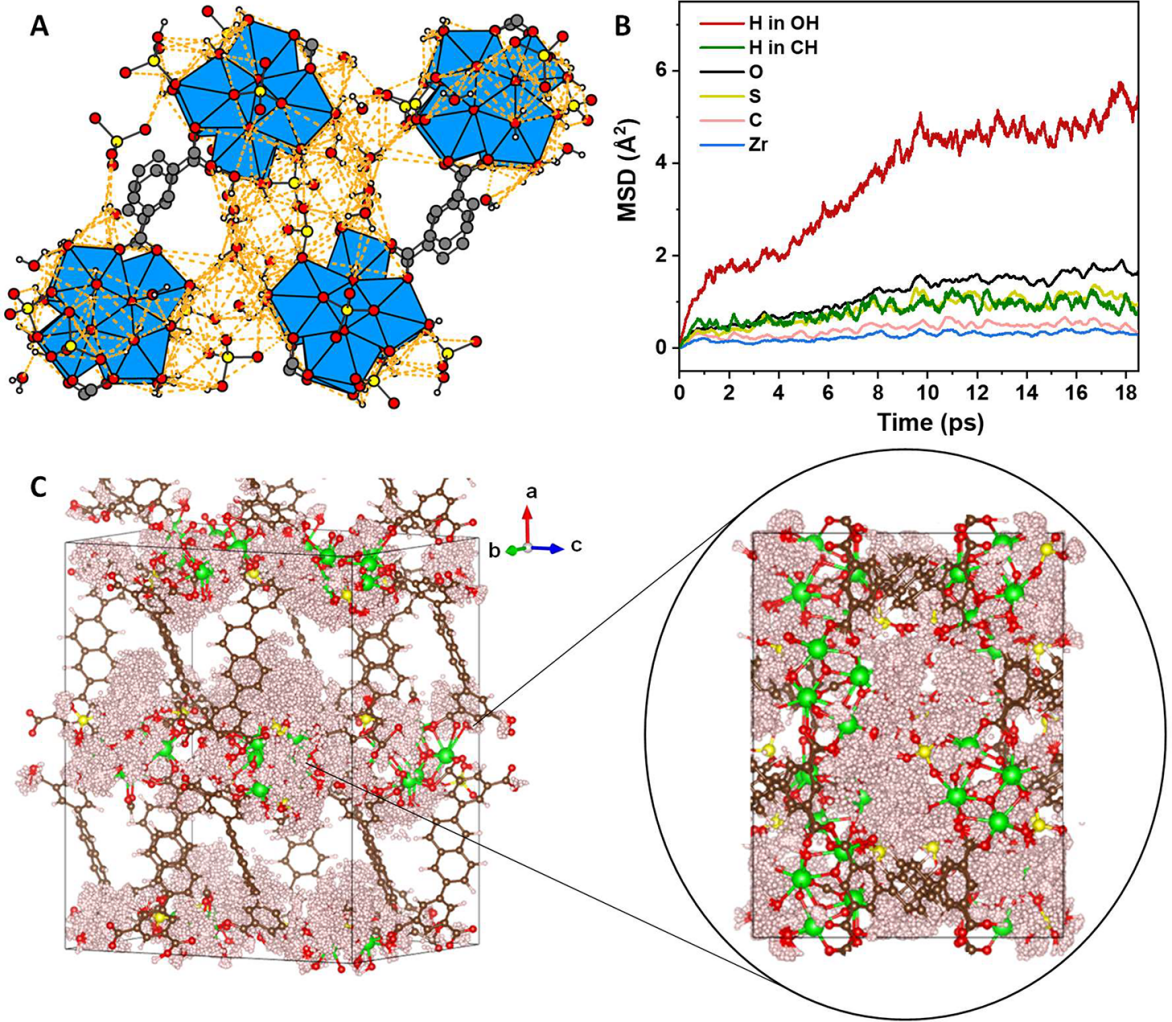
Structure of hydrogen-bonding network and MD simulation of proton transport in MFM-808-SO_4_. (A) View of potential hydrogen bonds (orange dashed lines; donor/acceptor distance ≤ 3.5 Å; *Ĥ* angle: 100–180°) among adjacent {Zr_6_} clusters within the *bc* plane. (Zr_6_, blue; O, red; S, yellow; C, gray; and H, white). (B) Mean square displacement as a function of time derived from MD simulations and (C) corresponding MD trajectories (H, misty rose; O, red; C, brown; Zr, green; and S, yellow). The view of the 2D conducting network along axis *a* is shown in the circle.

Replacing coordinated formates in MFM-808 with sulfates to give MFM-808-SO_4_ reduces the distance between the {Zr_6_}_2_ pairs within one layer from 3.4–4.8 to 1.5–2.6 Å as well as increasing the proton donor/acceptor sites. Solid-state NMR spectroscopic and potentiometric acid–base titration experiments reveal that the sulfates are protonated and in the form of HSO_4_^–^ with a low p*K*_a_ value of 2.60 ± 0.03 (Figures S15 and S16 and Table S3), indicating the presence of strong Brønsted acidic sites that can act as abundant proton sources. The bridging sulfate and −OH/H_2_O species are adjacent to each other (O···O = ∼1.5–5 Å) within {Zr_6_}-layers (Figure S24) and form extensive hydrogen-bonding networks to afford efficient pathways for protons transport within layers (Figures 3A and S25).

To gain further insights, a sample of MFM-808-SO_4_ with a lower loading of sulfates (5.7% S; denoted as MFM-808-SO_4_-LL, LL = low loading) was prepared to give a material that is deficient in sulfate species. MFM-808-SO_4_-LL shows an excellent proton conductivity as high as 6.42 × 10^–2^ S·cm^–1^ at 85 °C and 99% RH, *E*_a_ = 0.18 eV (Figures S26–S28). The detailed proton-conducting processes were analyzed by MD simulations which confirmed that protons from hydrosulfate/–OH/H_2_O centers were the only species that experienced diffusive movements (Figure 3B, Supporting Film). The MD trajectories further suggested that such diffusive displacement of protons is confined on and within the {Zr_6_} layer (Figure 3C), leading to an efficient 2D proton-conducting network. Proton hopping through the hydrogen-bonding networks can also be observed clearly in the simulation, which vividly illustrates the nature of Grötthuss mechanism in MFM-808-SO_4_ materials. Therefore, the excellent proton-conducting properties observed for the MFM-808-SO_4_ materials can be explained by the presence of rich proton sources and the available efficient 2D proton-transfer pathway. Such a 2D proton-conducting network on the {Zr_6_}-layer is reported here for the first time, and confirms that 2D MOF materials are capable of showing high proton conductivity. Moreover, MD simulations also indicated that the proton-conducting layers can be partially linked by the diffusion of adsorbed sulfate species and water molecules to provide additional proton transport pathways in the fully sulfonated material, MFM-808-SO_4_ (Figure S29), contributing to further improvement in the proton conduction performance (up to 0.21 S·cm^–1^). This is consistent with the values for *E*_a_ obtained from impedance analysis, where MFM-808-SO_4_ shows a slightly higher value for *E*_a_ (0.22 eV) compared with MFM-808-SO_4_-LL (0.18 eV). This reflects the additional energy barrier originating from the required reorientation or translation of adsorbed sulfate species and water molecules in the pores of MFM-808-SO_4_ during proton transfer.^29,30^ Nevertheless, such a barrier can be offset by the presence of abundant proton sources, which leads to an enhancement in the overall performance, as observed.^31^

### Photocatalytic HER

**Figure 4:**
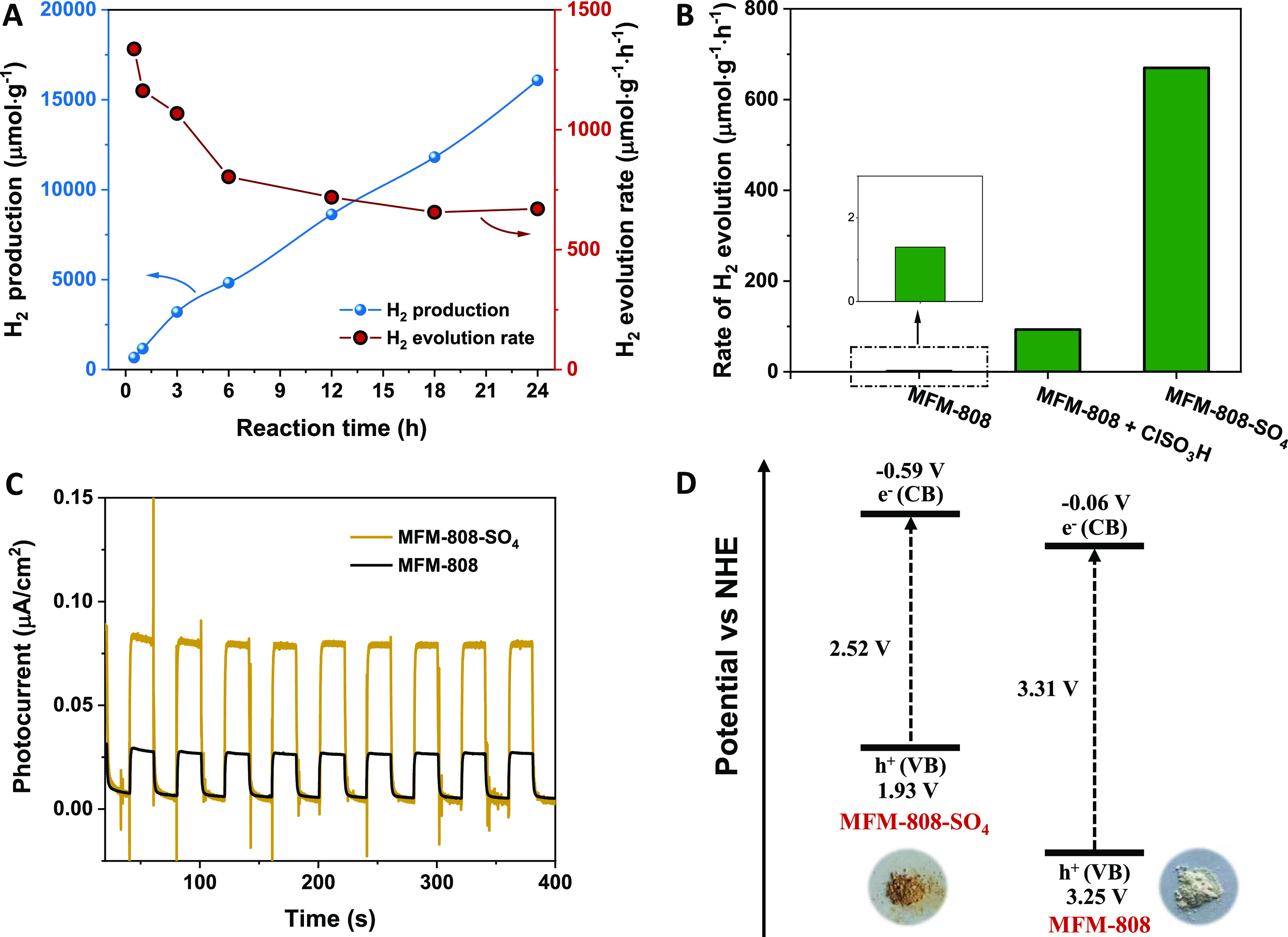
Photocatalytic HER performance. (A) Time-dependent H_2_ production and rate of production for MFM-808-SO_4_. (B) Average rate of hydrogen evolution rate catalyzed by MFM-808, MFM-808 physically mixed with ClSO_3_H, and MFM-808-SO_4_, respectively, under visible light irradiation for 24 h. (C) Photocurrent responses and (D) potential energy diagrams of *E*_CB_ (conduction band) and *E*_VB_ (valence band) for MFM-808 and MFM-808-SO_4_.

Hydrogen is an essential feed for PEMFCs to convert chemical energy into on-board electrical energy.^32^ We found that MFM-808-SO_4_ can also act as a photocatalyst to produce H_2_*via* light-driven water splitting. *In situ* production of H_2_ is one of the most appealing approaches for the development of PEM non-stationary fuel cells since it reduces the high costs and challenges associated with the storage and transportation of external H_2_. This approach also promotes the sustainable use of natural resources with zero emission at the point of use in a cycle of solar–chemical–electrical energy.^33−36^ Photocatalytic H_2_ evolution catalyzed over MFM-808-SO_4_ was stable under irradiation with a total production of 16,080 μmol·g^–1^ at an average rate of 670 μmol·g^–1^·h^–1^ (Figure 4A) over 24 h. This is superior to the performance of most MOFs and Zr-based materials and is even comparable to those coupled with noble metals or semiconductors as co-catalysts (Tables S4 and S5). Sulfation of MFM-808 to give MFM-808-SO_4_ triggers a desirable absorption in the visible light region (Figure S30A) due to a p−π conjugation between the lone-pair electrons of HSO_4_^–^/SO_4_^2–^ with aromatic phenyl and/or naphthalene rings of the bridging ligands^37^ and also provides strong Brønsted acid sites to act as additional proton sources (Figure S16, Table S3). As expected, MFM-808 showed a negligible H_2_ productivity. Moreover, MFM-808 + ClSO_3_H (physically mixed) exhibited much lower HER rates of 93.1 μmol·g^–1^·h^–1^ (Figure 4B). These results demonstrate the importance of integrating the functional species to promote electron transfer and suppress electron–hole recombination to drive efficient photocatalytic HER in MOFs. This was further confirmed by the measurements of the photocurrent response (Figure 4C), highest occupied molecular orbital (HOMO)–lowest unoccupied molecular orbital (LUMO) band gap analyzes (Figure 4D), Tauc analysis (Figure S30B), Mott–Schottky analysis (Figure S31), luminescent quenching and time-resolved photoluminescence measurements (Figures S32 and S33, Table S6), and by electrochemical experiments (Figure S34). The HOMO–LUMO band gap energy calculated from *Tauc* plots follows the order of MFM-808-SO_4_ (2.52 eV) < MFM-808 (3.31 eV) (Figure 4D). Combining the *Tauc* and Mott–Schottky analyses, the position of valence bands (*E*_VB_*vs* NHE) was determined to be +1.93 V for MFM-808-SO_4_ and +3.25 V for MFM-808, consistent with the higher activity in photocatalytic HER for the sulfated samples.

## Conclusions

In summary, the synthesis and characterization of MFM-808 showing an unprecedented ***mfm*** topology and unique {Zr_6_}-cluster-based layered structure have been achieved. Sulfation *via* single-crystal-to-single crystal transformation of MFM-808 yields MFM-808-SO_4_, which shows exceptionally high proton conductivity of 0.21 S·cm^–1^ at 85 °C and 99% RH with a novel 2D proton-conducting mechanism. MFM-808-SO_4_ can also act as a photocatalyst for water splitting to hydrogen, thus coupling its joint properties of excellent proton conduction with photocatalytic hydrogen production to unlock the development of a new platform of H_2_-PEMFCs with “solar-chemical-electric-energy-conversion” to promote the Hydrogen Economy.

## Supporting Information Available

Synthesis procedure, characterization, impedance analysis, MD simulation, photocatalytic studies (PDF)
Analysis by MD simulations confirming the mobility of protons from and between hydrosulfate/–OH/H_2_O centers (MPG)

## Supplementary Material

ja3c03943_si_001.pdf

ja3c03943_si_002.mpg
