# Supplementary material for: Ultra-fast Proton Conduction and Photocatalytic Water Splitting in a Pillared Metal–Organic Framework
Source: J Am Chem Soc. 2023 Aug 22;145(35):19225–31. doi: 10.1021/jacs.3c03943 (PMC10485888; doi:10.1021/jacs.3c03943)
Supplement: Supplementary file 1 — ja3c03943_si_001.pdf [file ja3c03943_si_001.pdf]

Supplementary Information

Ultra-fast Proton Conduction and Photocatalytic Water Splitting in a Pillared Metal-Organic Framework

Jin Chen[1#], Bing An[1#], Yinlin Chen[1], Xue Han[1], Qingqing Mei[1], Meng He[1], Yongqiang Cheng[2], Inigo J. Vitorica-Yrezabal[1], Louise S. Natrajan[1], Daniel Lee[3], Anibal J. Ramirez-Cuesta[2], Sihai Yang[1,4]* and Martin Schröder[1]*

[1]Department of Chemistry, The University of Manchester, Manchester, M13 9PL, UK

[2]Neutron Scattering Division, Neutron Sciences Directorate, Oak Ridge National Laboratory, Oak Ridge, TN 37831, USA

[3]Department of Chemical Engineering and Analytical Science, University of Manchester, Manchester, M13 9PL, UK

[4]College of Chemistry and Molecular Engineering, Beijing National Laboratory for Molecular Sciences, Peking University, Beijing 100871, China

[#]These authors contributed equally to this work.

*Corresponding authors: Sihai.Yang@manchester.ac.uk; M.Schroder@manchester.ac.uk

Contents

1. Materials and Methods S5

1.1 Materials S5

1.2 Synthesis of 5,5'-(naphthalene-2,6-diyl)diisophthalic Acid S5

1.3 Synthesis of MFM-808 .... S5

1.4 Synthesis of MFM-808-SO_4 .... S6

1.5 General characterization S6

1.6 Structure determination S7

1.7 Impedance measurements S7

1.8 Molecular dynamics (MD) simulations S7

1.9 Visible light-driven Hydrogen Evolution Reaction (HER) S8

1.10 Determination of optical band gaps S8

1.11 Photophysical studies S8

1.12 Photoelectrochemical measurements S9

2. Supplementary Schemes S10

Scheme S1. Synthesis of 5,5'-(naphthalene-2,6-diyl)diisophthalic acid. S10

Scheme S2. Synthesis of MFM-808 and MFM-808-SO_4. .... S10

3. Supplementary Figures S11

Figure S1. The ^{1}H NMR spectrum of the H_4L ligand in DMSO-d_6 .... S11

Figure S2. Optical microscopy of single crystals of MFM-808 and MFM-808-SO_4. .... S11

Figure S3. SEM images of MFM-808 and MFM-808-SO_4. .... S12

Figure S4. SEM images and corresponding EDX mapping of MFM-808. .... S12

Figure S5. SEM image and corresponding EDX mapping of MFM-808-SO_4. .... S13

Figure S6. PXRD patterns simulated for MFM-808 based upon single crystal structure and for as-synthesized MFM-808 and MFM-808-SO_4. .... S13

Figure S7. View of the $\{Zr_6\}_2$ pairs and their alignment in the crystallographic *bc* plane in MFM-808. .... S14

Figure S8. View of $\{Zr_6\}_2$ pairs and their alignment in the crystallographic *bc* plane in MFM-808-SO_4. .... S15

Figure S9. View of the $\{Zr_6\}_2$ pairs in the *bc* plane in MFM-808. View of structure of MFM-808 along the crystallographic *c*-, *b*- and *a*-axes. S16

Figure S10. Structure of MFM-808 viewed along the crystallographic *c*- and *b*-axes. S17

Figure S11. N_2 sorption isotherm at 77 K and pore size distribution analysis for MFM-808. .... S18

Figure S12. FTIR spectra for MFM-808 and MFM-808-SO_4 in the range of 4000-600 cm^{-1}. S19

Figure S13. TGA profiles and derivative TGA plots for MFM-808. .... S19

Figure S14. TGA profiles and derivative TGA plots for MFM-808-SO_4....................................S20

Figure S15. Solid state MAS ^1H NMR spectra for MFM-808 and MFM-808-SO_4. ....S20

Figure S16. Acid–base titration curve of MFM-808-SO_4 and first derivative curve..................S21

Figure S17. N_2 sorption isotherms of MFM-808-SO_4 at 77 K. S22

Figure S18. Water vapour sorption isotherms for MFM-808 and MFM-808-SO_4 at 298 K.......S22

Figure S19. Impedance analysis for MFM-808 (RH dependence, 25 °C).................................S23

Figure S20. Impedance analysis for MFM-808 (temperature dependence). S24

Figure S21. Impedance analysis for MFM-808-SO_4 (RH dependence). S25

Figure S22. Impedance analysis for MFM-808-SO_4 (temperature dependence)........................S26

Figure S23. Proton conductivity as a function of RH for MFM-808 and MFM-808-SO_4. ....S27

Figure S24. View of adjacent {Zr_6} clusters in the *bc* plane, the connection between {Zr_6}$_2$ pairs in the bc plane and proton-transport pathways between {Zr_6} clusters in MFM-808-SO_4. ....S28

Figure S25. View of potential hydrogen bonds within the layers of {Zr_6} clusters in MFM-808-SO_4. And potential routes/accessible areas for proton hopping on the {Zr_6}-layer in the *bc* plane. S29

Figure S26. N_2 sorption isotherms of MFM-808-SO_4-LL at 77 K. S30

Figure S27. Impedance analysis for MFM-808-SO_4-LL (RH dependence). S31

Figure S28. Impedance analysis for MFM-808-SO_4-LL (temperature dependence). S32

Figure S29. MD trajectories in MFM-808-SO_4 along *a*- and *b*-axis. S33

Figure S30. UV-vis-DRS diffuse reflectance spectra and the Tauc plots for MFM-808 and MFM-808-SO_4..S34

Figure S31. Mott–Schottky plots for MFM-808 and MFM-808-SO_4..S35

Figure S32. Emission spectra and plots of I_0/I (λ_{em} = 366 nm) for MFM-808-SO_4. Proposed oxidative and reductive quenching path in photocatalysis.. ....S36

Figure S33. Normalized decay transients at 366 nm, 384 nm and 403 nm emission wavelength (using excitation at 375 nm) in an aqueous solution for the ligand and MFM-808-SO_4:...........S37

Figure S34. Linear sweep voltammetry of MFM-808 and MFM-808-SO_4. Potential energy diagram of H_2 production from water over MFM-808-SO_4. ....S38

4. Supplementary Tables...S39

Table S1. Single crystal data and structural refinement for MFM-808 and MFM-808-SO_4.......S39

Table S2. Comparison of MOFs with a proton conductivity of $>10^{-2}$ S.cm^{-1}.............................S40

Table S3. Calculated pK_a values for MFM-808-SO_4, UiO-66, UiO-67, NU-1000, and MOF-808. ....S41

Table S4. Comparison of the photocatalytic HER activity in MOFs. S42

Table S5. Comparison of the photocatalytic HER activity with Zr-based materials..................S43

Table S6. The lifetime of ligand, MFM-808-SO_4 and its homogeneous control samples in the aqueous solution. S44

5. Supplementary References S45

1. Materials and Methods

1.1 Materials

Zirconium dichloride oxide hydrate ($ZrOCl_2.H_2O$, 99.9%), chlorosulfonic acid (99%), formic acid (98%), 2,6-dibromonaphthalene and tetrakis(triphenylphosphine)-palladium(0) [$Pd(PPh_3)_4$] were purchased from Alfa Aesar, Sigma-Aldrich, Fisher Scientific, Scientific Laboratory Supplies and TCI, respectively. All chemicals were obtained from commercial suppliers and used without further purification.

1.2 Synthesis of 5,5'-(naphthalene-2,6-diyl)diisophthalic acid (H_4L)

5,5'-(Naphthalene-2,6-diyl)diisophthalic acid (H_4L) was synthesized following a reported method with slight modification for preparation at scale and higher yield.[1] A mixture of benzene-1,3-dicarboxyethylester-5-boronic acid (13.3 g, 50.0 mmol), 2,6-dibromonaphthalene (5.72 g, 20.1 mmol), K_3PO_4 (25.0 g, 117.8 mmol) and 1,4-dioxane (300 mL) was degassed under N_2 for 1 h. $Pd(PPh_3)_4$ (700 mg, 0.606 mmol) was added with stirring and the reaction heated to 80 °C under N_2 for 3 days. The resultant mixture was evaporated under reduced pressure. The tetra-ethyl ester of the target ligand was extracted with CH_2Cl_2 from the dry mixture. The CH_2Cl_2 solution was evaporated under reduced pressure and the residual solid washed with EtOH. The white solid was hydrolyzed by refluxing in 6 M NaOH overnight. The resulting solution was then filtered and the solution acidified via addition of 37% HCl to pH to 2. The yellow product was obtained by filtration and dried in air (yield: 78.8%). ^{1}H NMR (400 MHz, DMSO-d_6): δ 8.58 (t, J = 1.6 Hz, 2H), 8.52 (d, J = 1.6 Hz, 1H), 8.45 (d, J = 1.8 Hz, 1H), 8.25 (d, J = 8.6 Hz, 1H), 8.00 (dd, J = 8.6, 1.8 Hz, 1H).

1.3 Synthesis of MFM-808

$ZrOCl_2.H_2O$ (268.8 mg, 0.815 mmol), N,N'-dimethylformamide (DMF, 66.8 mL), formic acid (33.4 mL) were mixed and sonicated for 30 min. H_4L (100 mg, 0.220 mmol) was added and the mixture sonicated for 30 mins. The resulting solution was sealed in a Teflon-lined stainless-steel autoclave and heated at 130 °C for 2 days (CAUTION: Unsuitable containers, such as glassy pressure tube, can result in serious explosion!). The white solid product of MFM-808 was isolated by filtration. The product was washed with DMF and acetone (ten times for each) over a 1 week period. Elemental analysis, [$Zr_6O_4(OH)_{6.5}(H_2O)_2DMF(HCOO)_{5.5}L$]: found%(calc.%): Zr, 35.4(35.0); C, 25.6(26.5); H, 2.2(2.3); N, 0.4(0.9).

1.4 Synthesis of MFM-808-SO_4

MFM-808 (400 mg) was activated under dynamic vacuum at 130 °C overnight (sample mass changed to 260 mg, i.e., 0.17 mmol, after activation). The sealed system was charged with Ar and dry CH_2Cl_2 (20 mL) and stirred in an ice-water bath for 30 mins. Chlorosulfonic acid (200 μL, 3.0 mmol) was added to the mixture, which was reacted for 6 h under Ar. The temperature rose from 0 °C to room temperature during this period, and the resultant orange solid was separated and collected by centrifugation (8000 rpm, 1 min), washed with CH_2Cl_2 and acetone once each and dried in an oven to afford MFM-808-SO_4. Elemental analysis, $[Zr_6O_4(OH)_4(H_2O)_{8.4}(SO_4)_{2.5}L.(HSO_4)_3.(H_2O)_{8.3}]$, found%(calc.%): Zr, 26.7(27.7); C, 16.2(15.8); H, 2.6(2.6); N, 0(0); S, 9.4(8.9).

1.5 General characterization

Powder X-ray diffraction (PXRD) patterns were measured in the 2θ range from 3° to 50° on a Panalytical X'Pert MPD diffractometer with Cu-Kα (λ = 1.54056 Å, 40 kV and 30 mA). Elemental analysis was carried out on an FLASH 2000 elemental analyzer. Infrared spectra were collected in the range of 4000-500 cm^{-1} by a Thermo Scientific Nicolet iS5 spectrometer with iD5 ATR attachment. Scanning electron microscopy (SEM) images were obtained on Zeiss Scanning Electron microscope with an EDS7636 SiLi dectector for EDX measurement. Thermogravimetric analysis (TGA) was performed under air flow (100 $mL.min^{-1}$) at a heating rate of 10 °C min^{-1} from room temperature to 800 °C on a Pyris 1 Thermogravimetric Analyser (Perkin Elmer). Solution ^1H NMR spectra were collected on a B400 Bruker Avance III 400 MHz. UV-Vis diffuse reflectance spectra were collected in the region 1400 to 200 nm on a Shimadzu UV-2600 spectrophotometer equipped with an integrating sphere utilising $BaSO_4$ as reference.

BET surface areas were calculated from N_2 isotherms collected at 77 K on a Micromeritics 3-flex apparatus. Samples were activated at 10^{-10} bar by heating at 130 °C overnight before measurements. Pore size distribution was derived from N_2 isotherms using non-local density functional theory applied within the 3-flex software package. Magic angle spinning (MAS) NMR spectra were recorded using a Bruker 9.4 T (400 MHz ^1H Larmor frequency) AVANCE III spectrometer equipped with a 4 mm HFX MAS probe. Samples were treated and packed into 4 mm o.d. zirconia rotors under inert gas and sealed with a Kel-F rotor cap. Experiments were acquired at ambient temperature using a MAS frequency of 12 kHz. ^1H pulses of 100 kHz

were used for the ^1H MAS NMR spectra that employed a Hahn-echo sequence with an inter-pulse delay of one rotor period, giving a total echo time of 0.167 ms. 16 Transients were co-added for each spectrum, with a recycle delay of $1.3T_1$ (~3–4 s) used between the scans.

1.6 Structure determination

Single crystal X-ray diffraction data were collected at 100 K on a Rigaku FR-X diffractometer equipped with an Oxford Cryosystems liquid nitrogen flow system using CuKα radiation (λ = 1.5418 Å) and a CCD detector. Data collection, frame integration and data processing were performed using CrysAlisPro program suite.[2] The structure was solved by direct method and refined by full-matrix least-squares on F^2 with the anisotropic approximation (for non-hydrogen atoms) using Olex2 software package.[3] Hydrogen atoms of the organic ligands were calculated geometrically and refined by a riding model. The crystallographic data and structural refinement are shown in Table S1.

1.7 Impedance measurements

Proton conductivity was measured by impedance analysis performed by a Solatron SI 1260 Impedance/Gain phase Analyzer over a frequency range of 1~10^6 Hz with an amplitude of 100 mV and 0 mV DC rest voltage. The samples were finely ground and pressed at 5 tons pressure into pellets of 8 mm in diameter and ~0.6 mm in thickness and coated with silver paste on both sides to improve the contact with the platinum electrodes in an electrochemical cell. The temperature and relative humidity (RH) in these experiments were regulated by a Kambic KK-50 climatic chamber and monitored using a Rotronic HC2-C04 probe. Proton conductivity (σ, $S.cm^{-1}$) was obtained using the equation σ = l/(RS), where l and S are the thickness and cross-sectional area of sample pellets, respectively, and R is the resistance of the sample analyzed from Nyquist plots using ZView software. The activation energy (E_a) was extracted from measurements of σ over various temperatures at 99% RH using the *Arrhenius* equation: $\sigma = (\sigma_0/T)\exp[-E_a/(kT)]$, where σ_0, T and k are the pre-exponential factor, temperature in K and Boltzmann constant, respectively.

1.8 Molecular dynamics (MD) simulations

MD simulations were performed using CP2K (http://www.cp2k.org)[4] based on the mixed Gaussian and plane-wave scheme[5] and the Quickstep module.[6] The calculation used molecularly optimized Double-Zeta-Valence plus Polarization (DZVP) basis set,[7] Goedecker-

Teter-Hutter pseudopotentials,[8] and the Perdew-Burke-Ernzerhof (PBE) exchange correlation functional.[9] The plane-wave energy cutoff was 400 Ry. The DFT-D3 level correction for dispersion interactions, as implemented by Grimme et al,[10] was applied with a cutoff distance of 15 Å. Structural optimization was performed using the Broyden-Fletcher-Goldfarb-Shannon (BFGS) optimizer prior to the MD simulation. An NVT ensemble was simulated with T= 400 K and time-step of 0.5 fs. After equilibrium, a trajectory of over 37,000 steps (18.5 ps) was recorded and then analyzed. Note that DFT calculations for the large unit cell in this case (832 atoms) is computationally intensive and therefore an elevated temperature was used in MD simulation to accelerate the dynamics.

1.9 Visible light-driven Hydrogen Evolution Reaction (HER)

Photocatalytic HER was carried out in an external illumination type reaction vessel with a magnetic stirrer. 5.0 mg of sample was dispersed in 10 mL septum-sealed glass vials. Each sample was made up to a volume of 2 mL including 1.9 mL CH_3CN, 0.1 mL H_2O, and 0.1 mL CH_3OH as the sacrificial agents. Sample vials were capped and deoxygenated by bubbling with Ar for 10 mins to ensure complete removal of air. The solution was irradiated using a Xenon light source with a 400 nm filter. After the reaction, the gas in the headspace of the vial was analyzed by GC to determine the amount of H_2 generated. No CO_2 or CO was detected in this study.

1.10 Determination of optical band gaps

The optical band gaps (E_g) were calculated using the Kubelka–Munk equation and a Tauc plot, which was calculated by replacing molar absorption coefficient (α) with absorbance (Abs) in equation, $(\alpha h\nu)^2=A(h\nu-E_g)$, where α, h, ν, A and E_g are the molar absorption coefficient, Plank's constant, frequency, absorbance and band gap, respectively.[11]

1.11 Photophysical studies

Steady-state emission spectra were measured on an Edinburgh Instrument FP920 Phosphorescence. Emission wavelengths were selected with band-pass filters (Semrock and Chroma). For consistency and to ensure reproducibility, steady-state luminescence experiments were carried out using identical instrumental settings and all reported data are an average of at least 3 runs. The quenching experiments were supported by fitting obtained intensities at 610 nm to the concentration using the Stern-Völmer equation:

$$\frac{I_0}{I} = 1 + K_{SV}C$$

where K_{SV} is the Stern-Völmer constant and I_0/I is the ratio of luminescence intensity in the absence and presence of $\{Zr_6\}$ clusters or CH_3OH.

Time-domain lifetimes were measured using the Time-Correlated Single Photon Counting (TCSPC) method on a Lifetime Spectrometer equipped with a 5 W microsecond pulsed Xe flash lamp, a 450 W steady state Xe lamp, and a Hamamatsu R928P red-sensitive photomultiplier in a peltier, air cooled housing (Hamamatsu R928P). The fluorimeter incorporated Becker-Hickl SPC-130 detection electronics and a HPM-100-40 Hybrid PMT detector. All spectra were corrected for the detector response and excitation source using the instrument correction files. Lifetimes were obtained by the tail fit on the obtained data, and the quality of fit was judged by minimization of reduced chi-squared and residuals squared parameters.

1.12 Photoelectrochemical measurements

Electrochemical measurements were conducted in a standard three-electrode glass cell connected to a CHI760e electrochemical workstation. A 3-electrode system was used with a platinum plate as the counter electrode and Ag/AgCl as a reference electrode. The as-prepared sample was coated on a glassy carbon disk (3 mm diameter disk) and functioned as the working electrode.

Mott–Schottky (MS) plots of samples were measured on an electrochemical workstation (Zahner Zennium). A 0.1 M Na_2SO_4 solution was used as the electrolyte. The catalyst (~2 mg) was added to 2 mL of EtOH containing 10 μL 5 wt% Nafion, and the working electrode was prepared by dropping the mixed suspension (30 μL) onto the surface of the glassy carbon electrode. MS plots were measured at different frequencies to determine the position of the conduction band (CB). The MS plots gave positive slopes for the linear plots and confirm typical *n*-type semiconductor characteristics; a negative slope is expected for a *p*-type semiconductor (Fig S31).[12]

Photocurrent measurements were also performed using a three-electrode system with the photocatalyst-coated into FTO (Fluorine-doped Tin Oxide) (4 cm^2) as the working electrode, a Pt plate as the counter electrode, and a Ag/AgCl as reference electrode. A 300 W Xe lamp with a cut-off filter ($\lambda > 400$ nm) was used as light source.

2. Supplementary Schemes

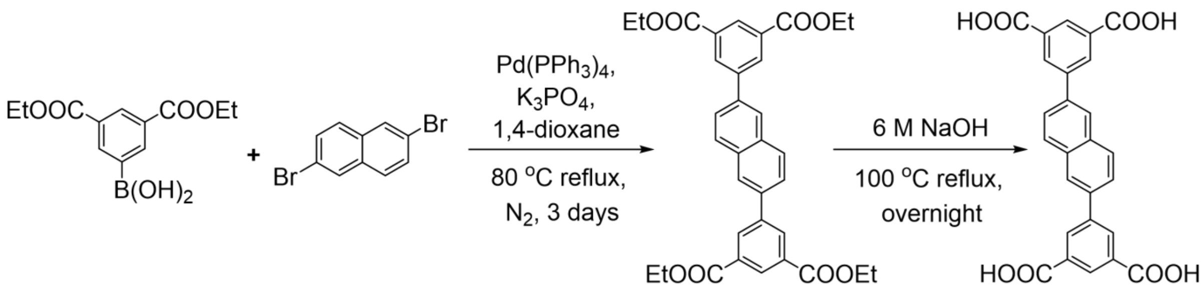

Scheme S1. Synthesis of 5,5'-(naphthalene-2,6-diyl)diisophthalic acid (H_4L).

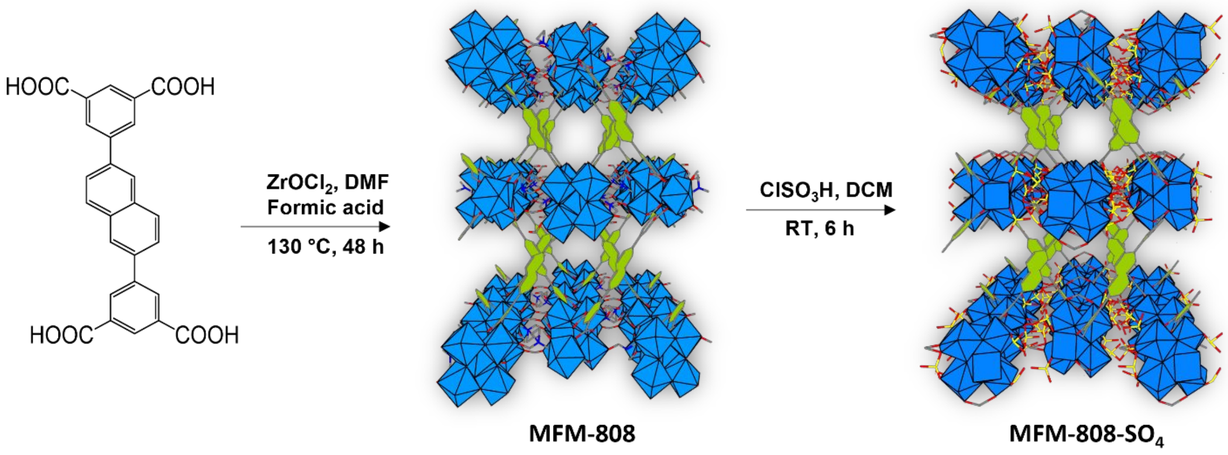

Scheme S2. Synthesis of MFM-808 and MFM-808-SO_4 (RT = room temperature).

3. Supplementary Figures

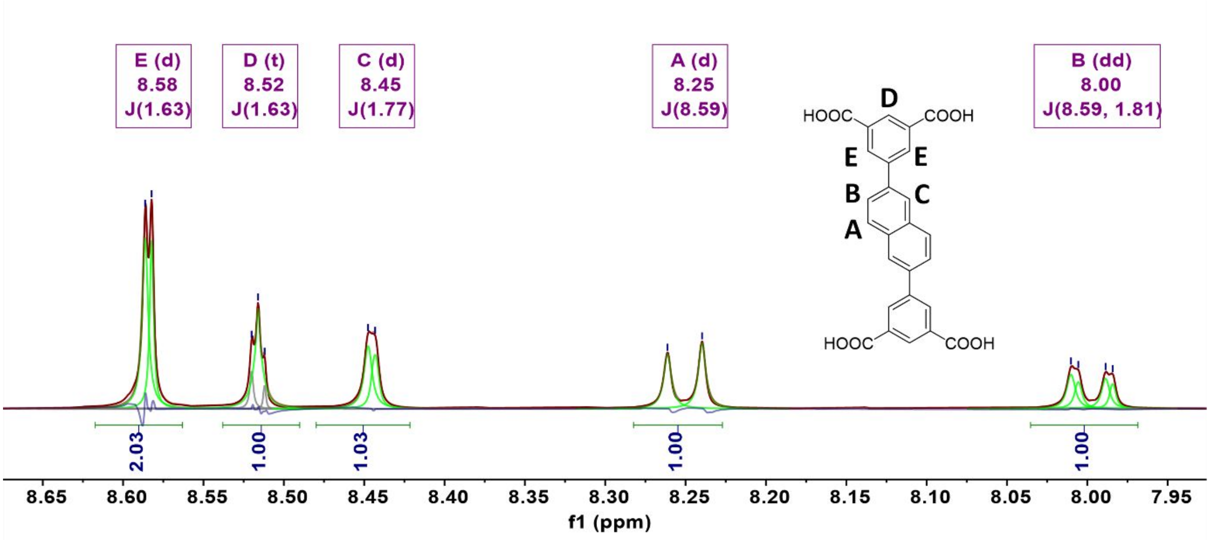

Figure S1. The ^1H NMR spectrum of the H_4L ligand in DMSO-d_6.

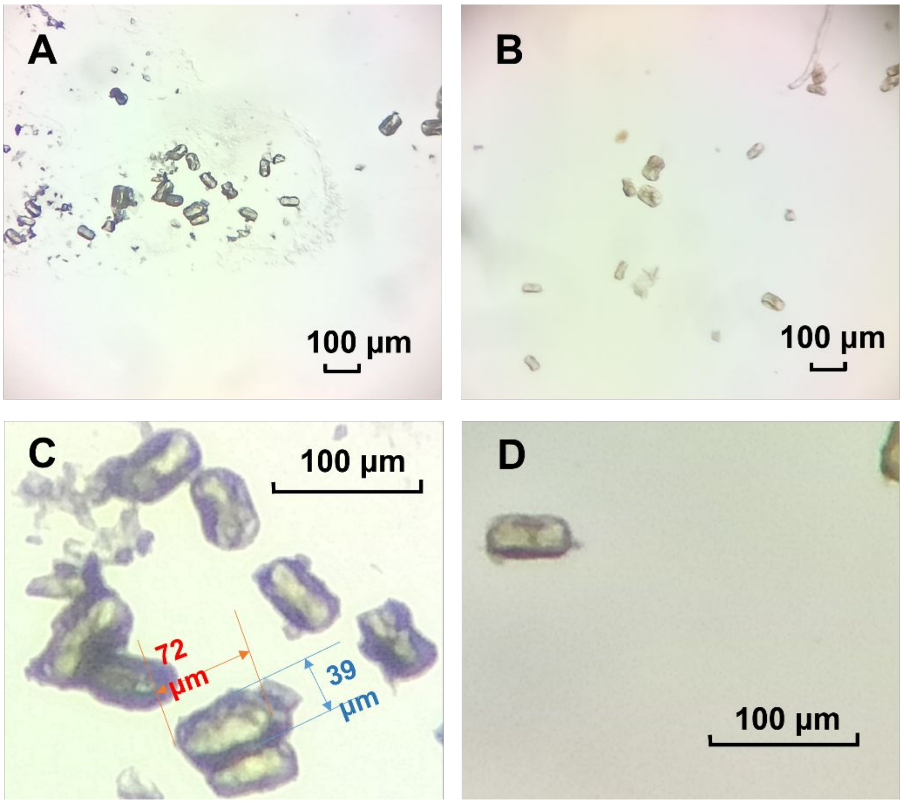

Figure S2. Optical microscopy of single crystals of (A) MFM-808, (B) MFM-808-SO_4 with a scale bar of 100 µm. Zoom-in views of (C) MFM-808 and (D) MFM-808-SO_4.

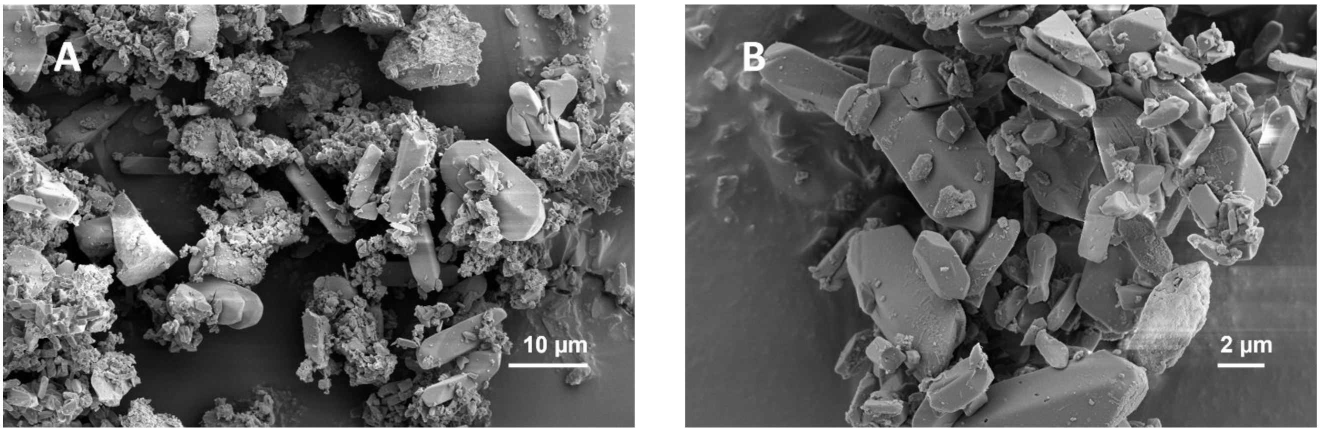

Figure S3. SEM images for (A) MFM-808 and (B) MFM-808-SO_4.

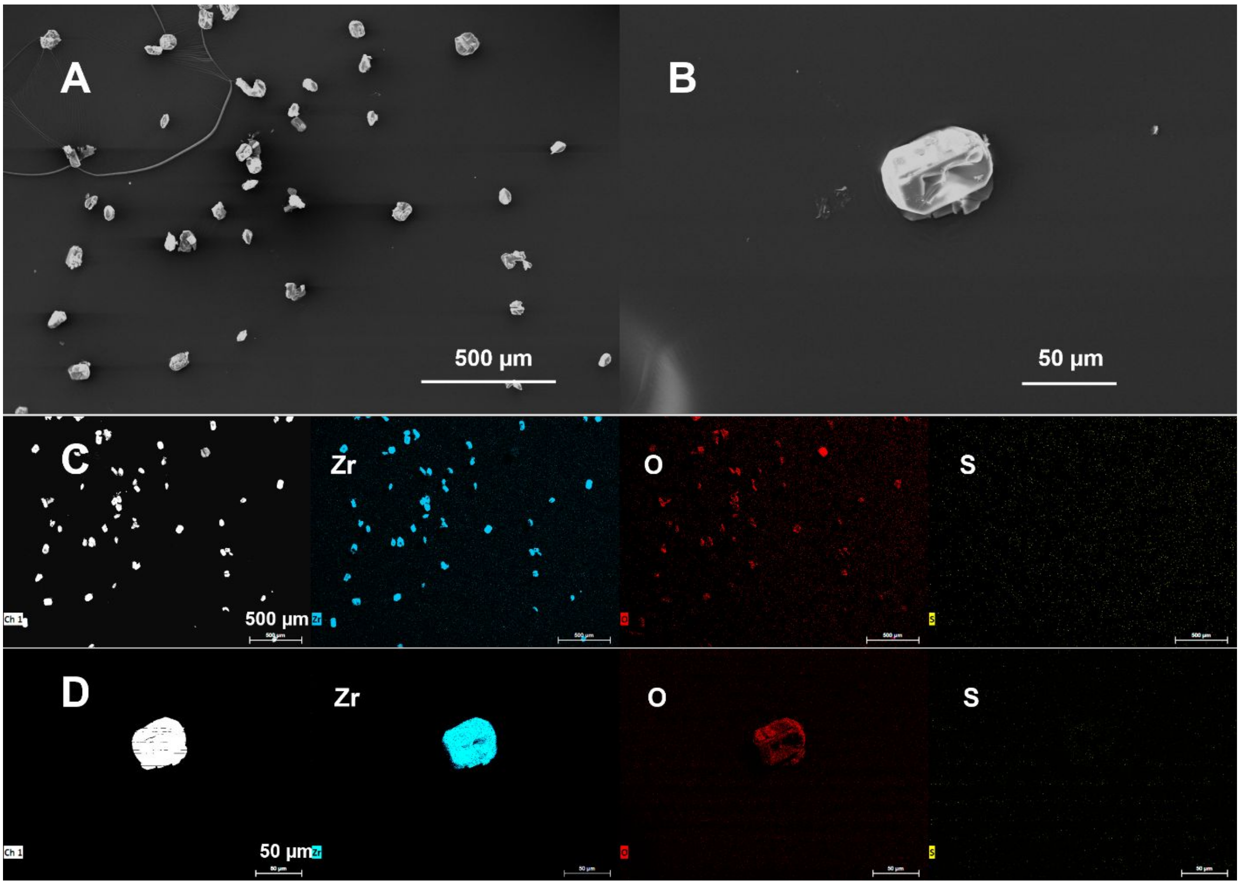

Figure S4. SEM image of MFM-808 with scale bar of (A) 500 μm and (B) 50 μm; corresponding EDX mapping for chromium, oxygen and sulfur with scale bar of (C) 500 μm and (D) 50 μm.

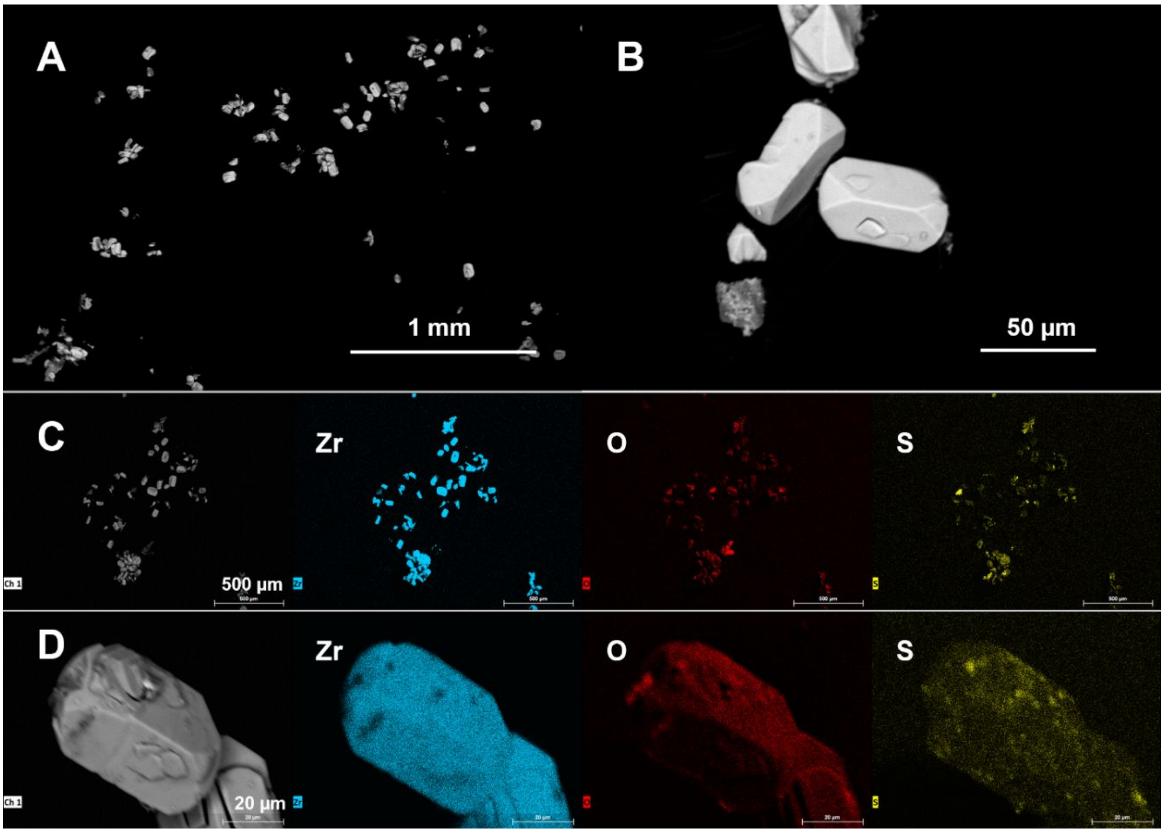

Figure S5. SEM image of MFM-808-SO_4 with scale bar of (A) 1 mm and (B) 50 µm; corresponding EDX mapping for chromium, oxygen and sulfur with scale bar of (C) 500 µm and (D) 20 µm.

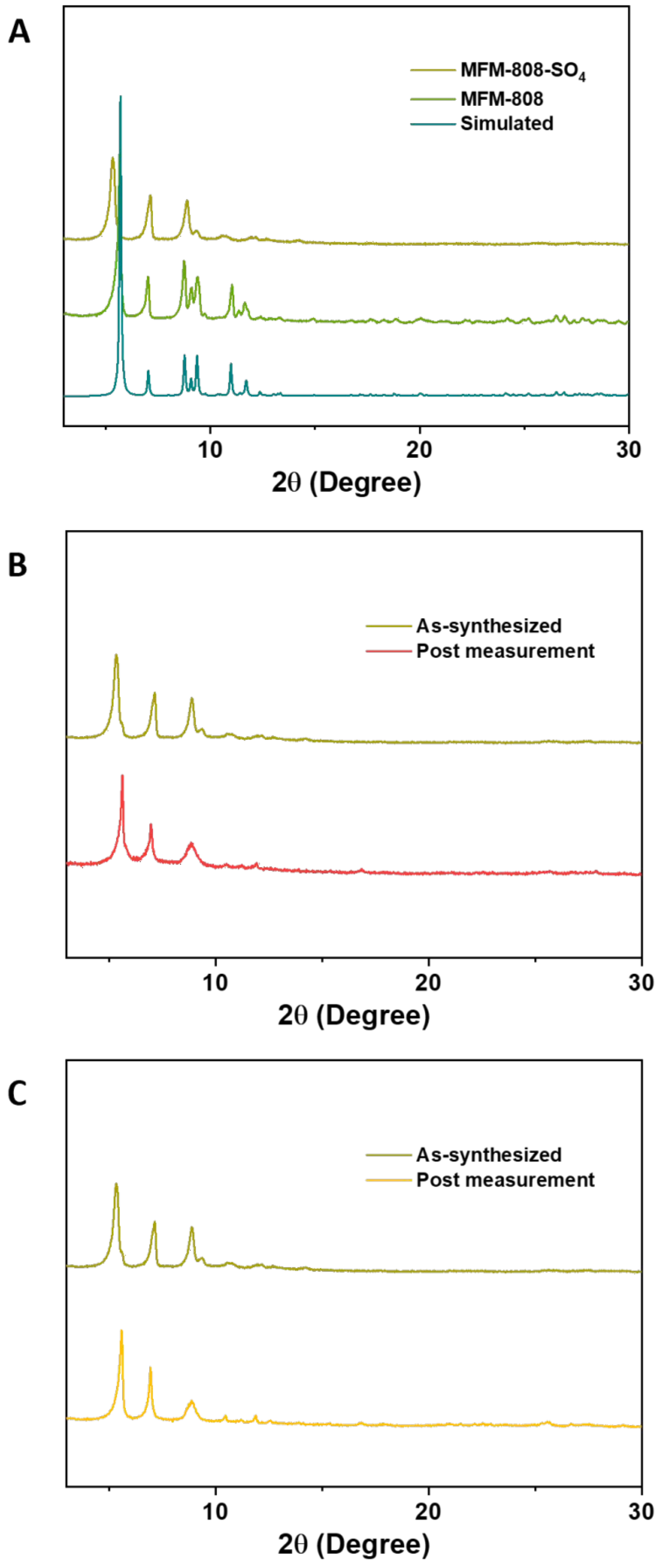

Figure S6. (A) PXRD patterns for simulated for MFM-808 based upon single crystal structure and for as-synthesized MFM-808 and MFM-808-SO_4. (B) PXRD patterns for as-synthesized MFM-808-SO_4 and MFM-808-SO_4 after impedance measurements. (c) PXRD patterns for as-synthesized MFM-808-SO_4 and MFM-808-SO_4 after photocatalytic measurements.

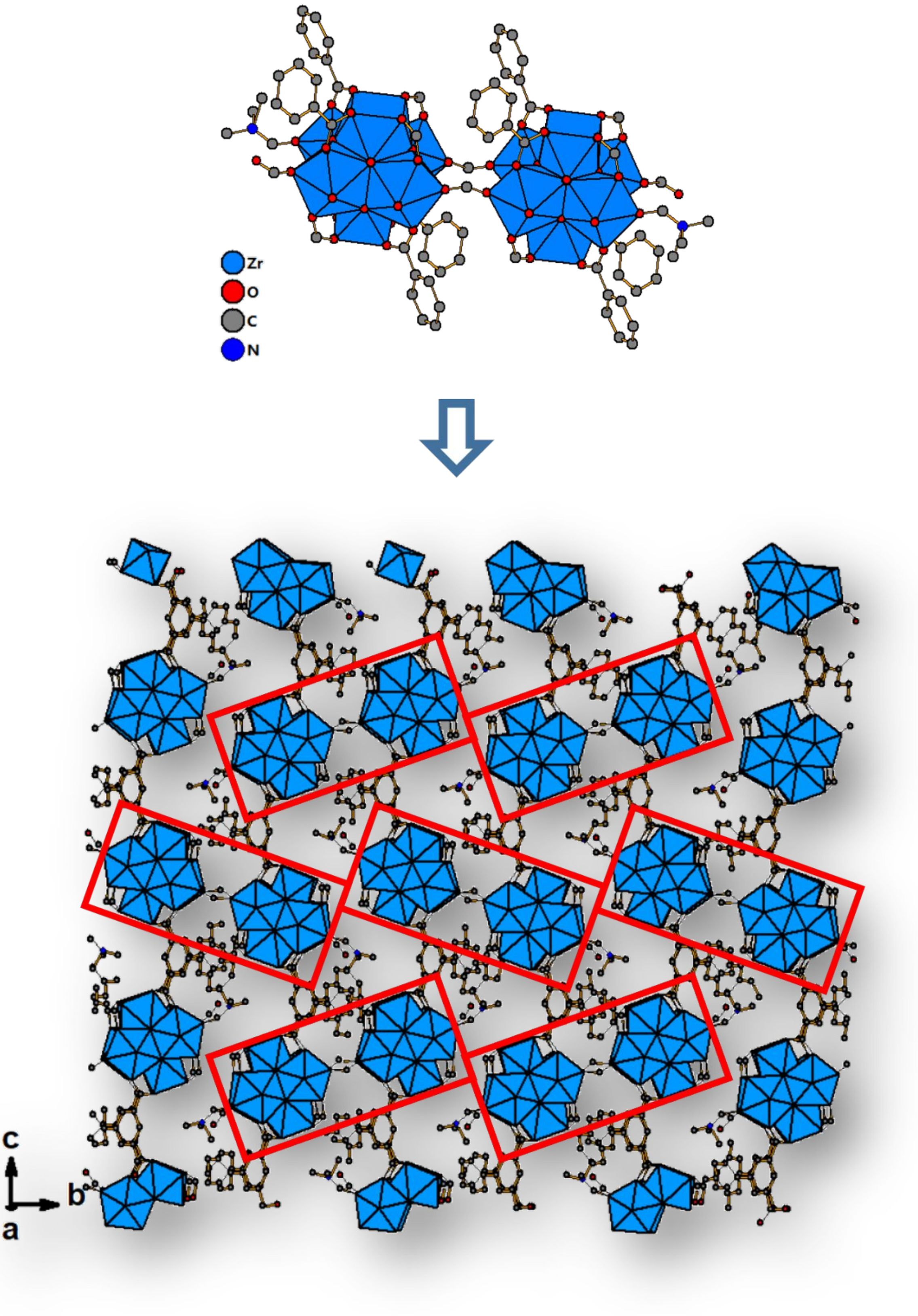

Figure S7. View of the $\{Zr_6\}_2$ pairs and their alignment in the crystallographic *bc* plane in MFM-808.

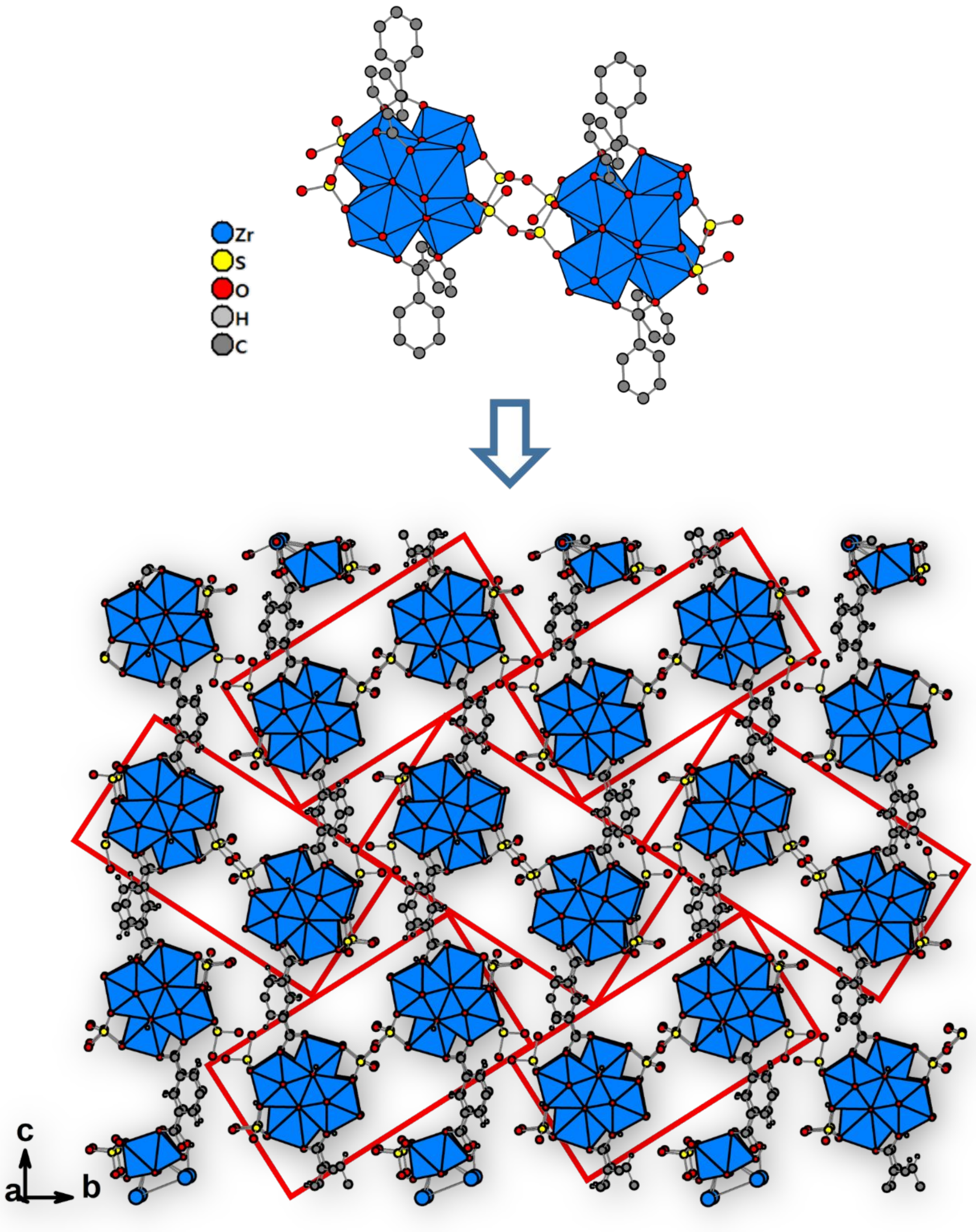

Figure S8. View of $\{Zr_6\}_2$ pairs and their alignment in the crystallographic *bc* plane in MFM-808-SO_4.

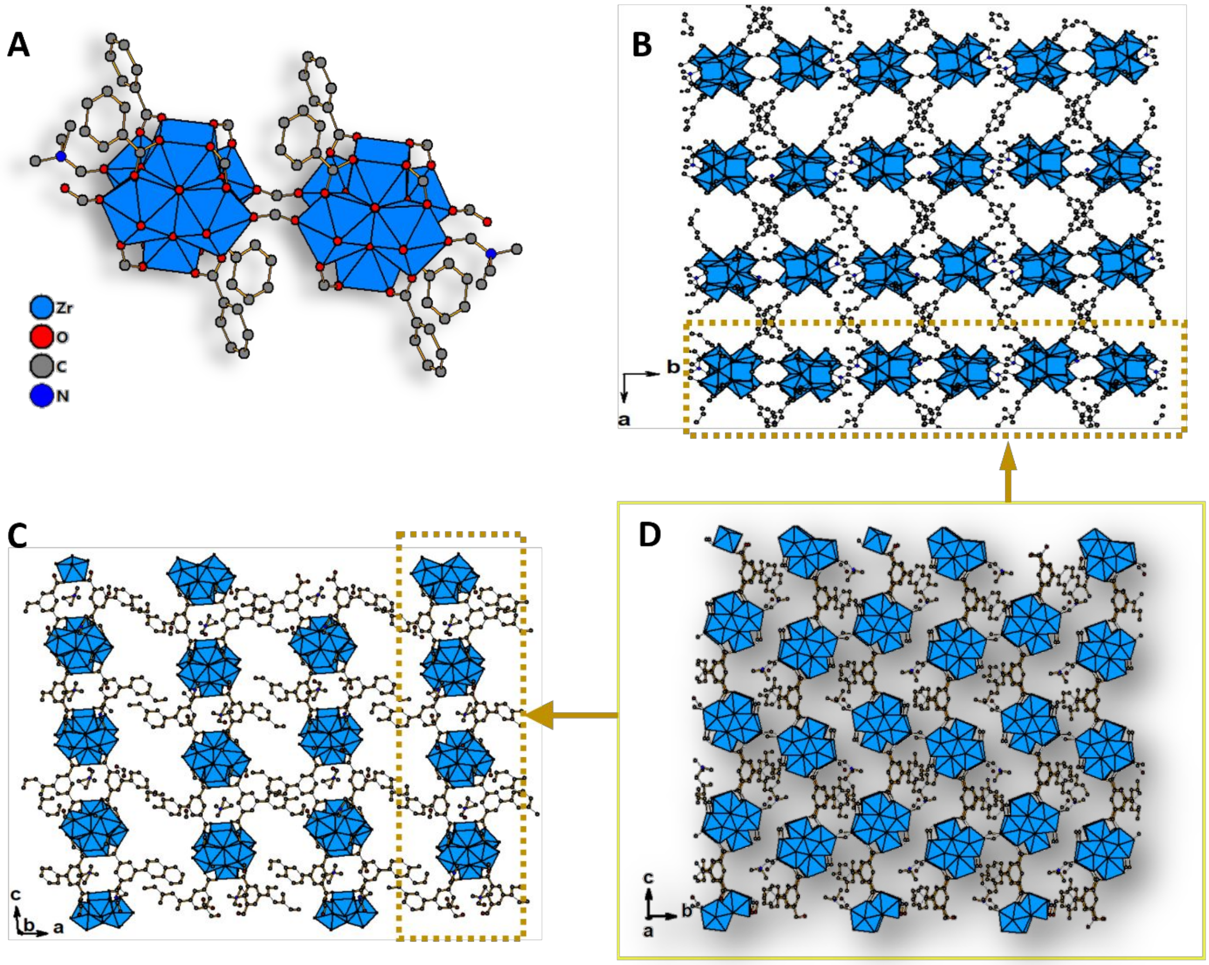

Figure S9. (A) View of the $\{Zr_6\}_2$ pairs in the *bc* plane in MFM-808. View of structure of MFM-808 along the crystallographic (B) *c*-, (C) *b*- and (D) *a*-axes.

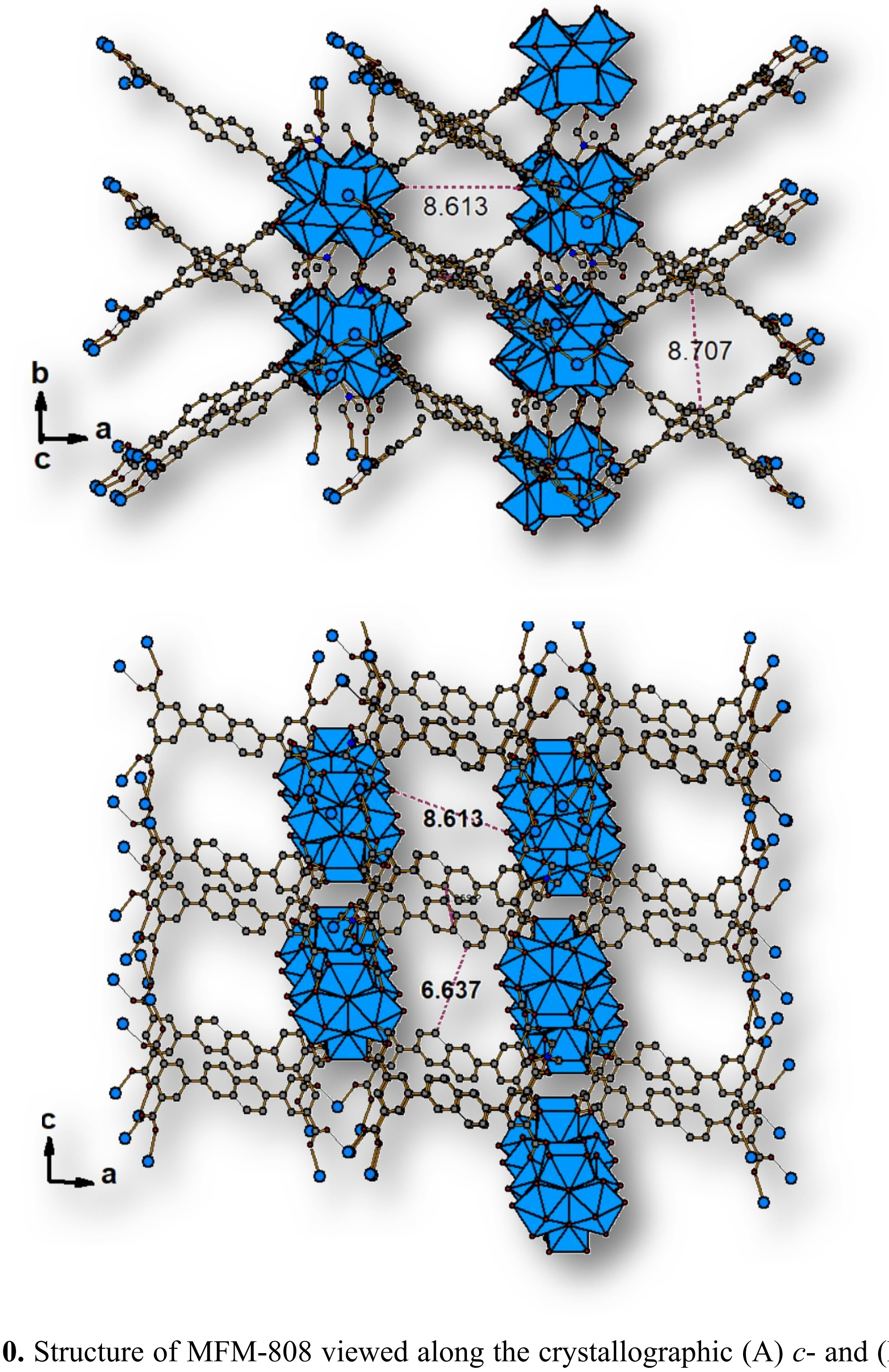

Figure S10. Structure of MFM-808 viewed along the crystallographic (A) *c*- and (B) *b*-axes. The distances are in Å.

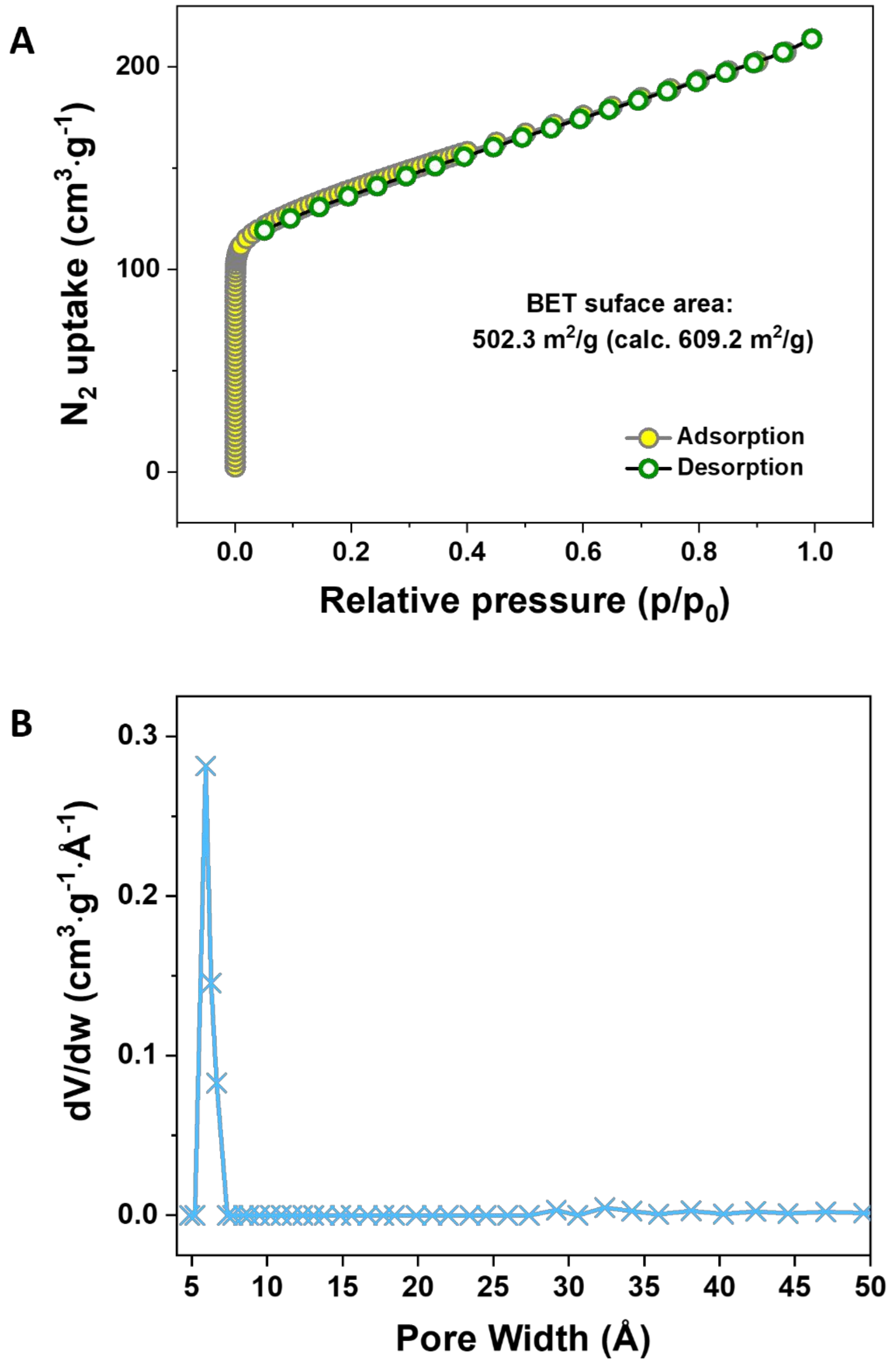

Figure S11. (A) N_2 sorption isotherm at 77 K and (B) corresponding pore size distribution (PSD) analysis for MFM-808. The BET surface area was calculated to be 502 $m^2.g^{-1}$, close to the theoretical value (609 $m^2.g^{-1}$) based upon the single crystal structure as calculated using the reported method.[13]

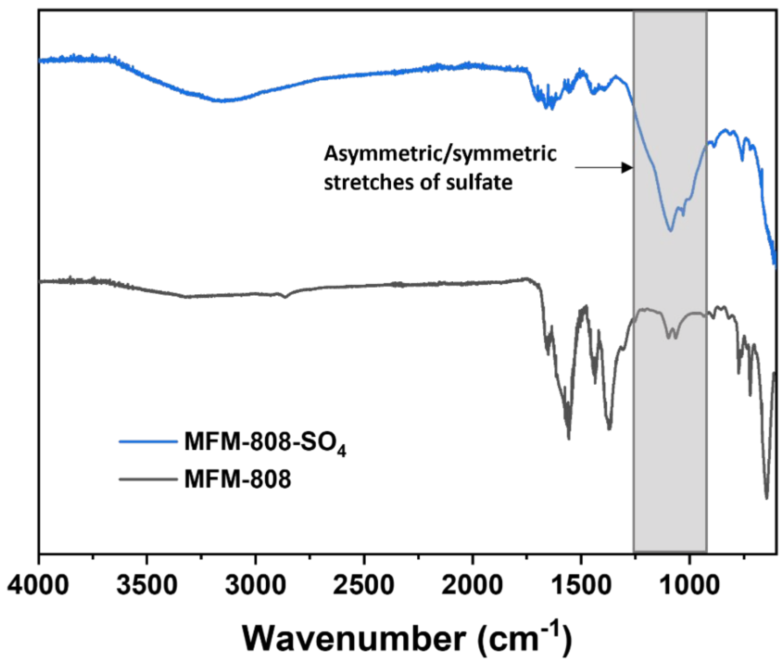

Figure S12. FTIR spectra for MFM-808 and MFM-808-SO_4 in the range of 4000-600 cm^{-1}.

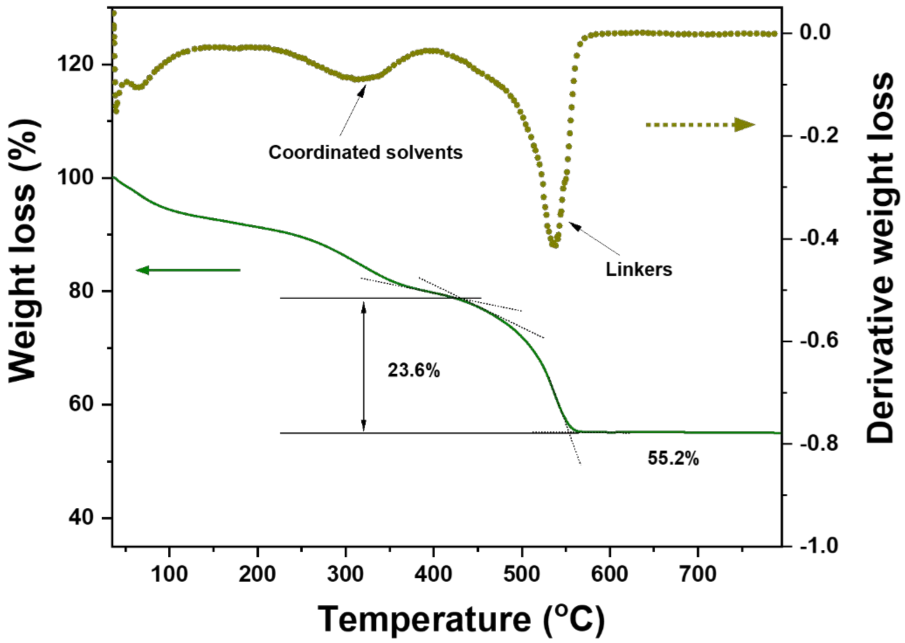

Figure S13. TGA profiles and derivative TGA plots for MFM-808.

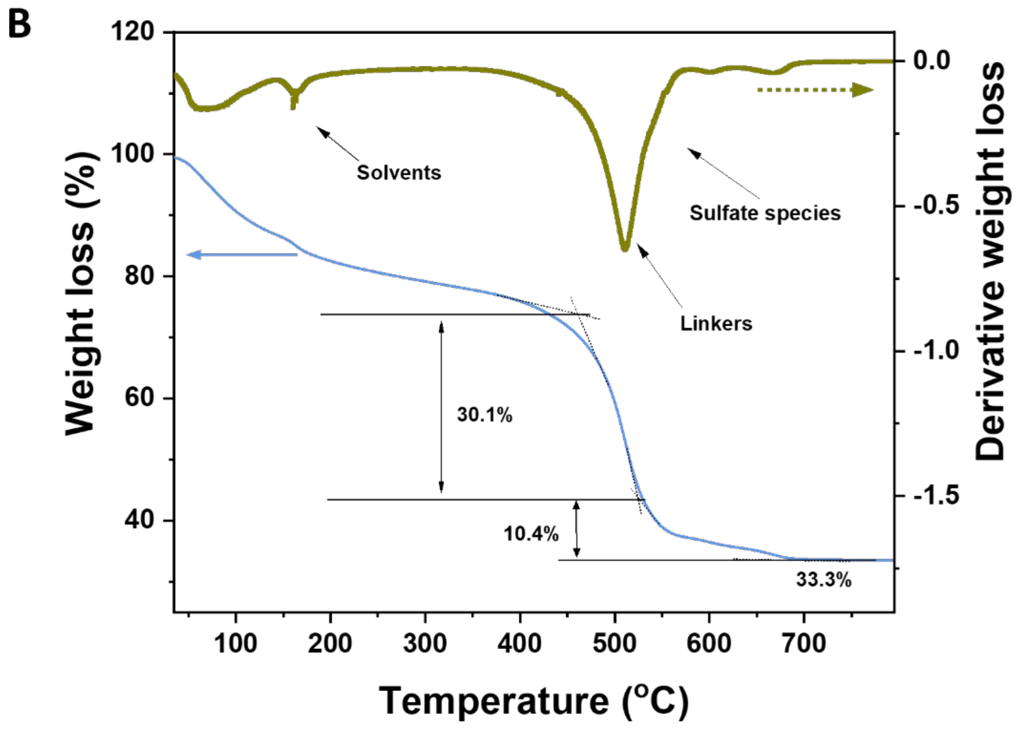

Figure S14. TGA profiles and derivative TGA plots for MFM-808-SO_4.

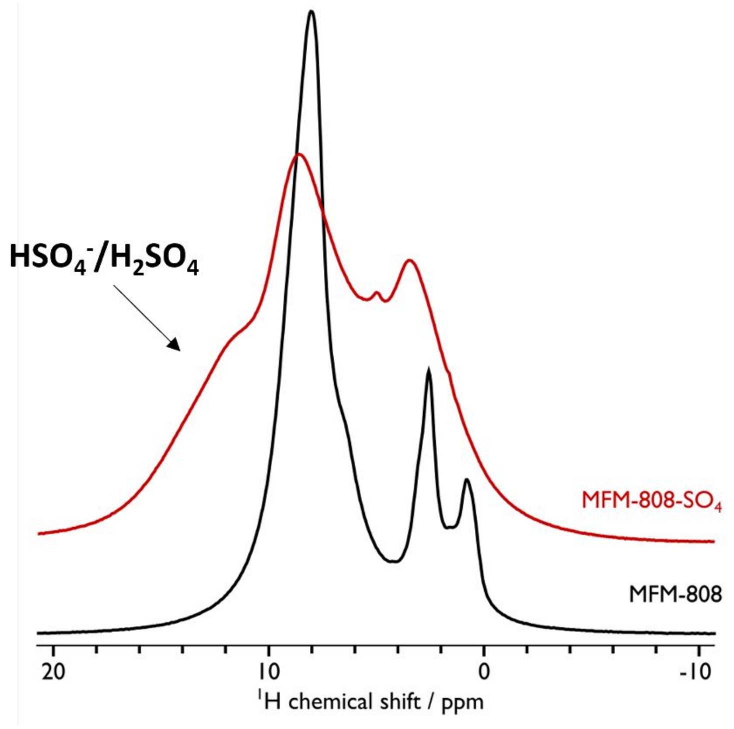

Figure S15. Solid state MAS ^1H NMR spectra for MFM-808 and MFM-808-SO_4.

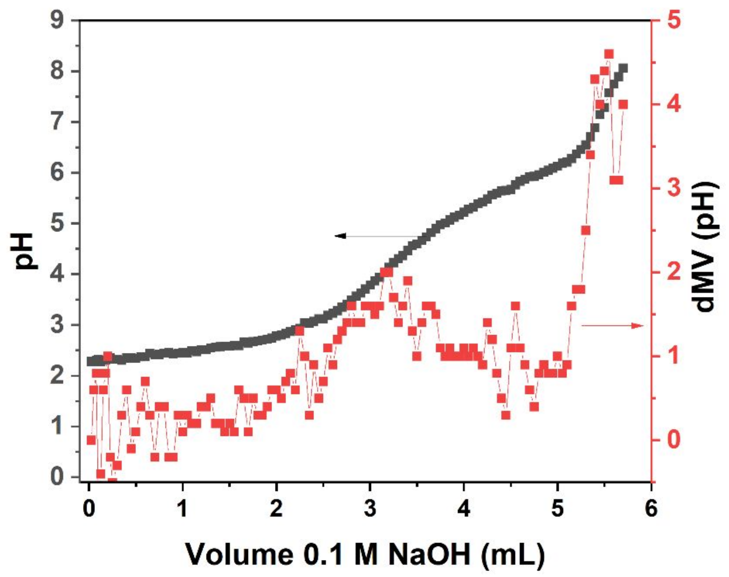

Figure S16. Acid–base titration curve of MFM-808-SO_4 (black) and first derivative curve (red).

Potentiometric acid–base titration experiments were conducted to quantify the concentration of Brønsted acid sites. MFM-808-SO_4 was dispersed in an aqueous solution of $NaNO_3$ for 15 h in order to exchange ions. The solid was removed and the resulting solution titrated with 0.1 M aqueous NaOH to pH = 8.0. Sufficient time had to be given for the pH to stabilise during the titration process, and typically ~1 mL addition of NaOH required at least 1 h to reach a stable pH value.

Such titration experiment can not only give information about local Brønsted acidity in the sample, but also shows insights into the identity and number of defect sites. As shown in Figure S16, two equivalence points at pH = 3.9 ± 0.3 and pH = 7.2 ± 0.4 were observed in the titration curve for MFM-808-SO_4, giving the pK_a value of pK_{a1} = 2.60 ± 0.03 and pK_{a2} = 3.41 ± 0.06, respectively. We assign these to two types of titratable protons derived from HSO_4^- and μ_3-OH (Table S3), on the framework.

We also titrated the filtrate after removal of the solid MOF. As this method was based on the kinetic equilibrium of cation-exchange between Na^+ and H^+, the results can only reflect those Na^+ accessible acidic sites with low pK_a values on the framework. As a result, the concentration of acidic protons of the filtrate was determined as 10.3 ± 0.4 mol·mol_{MOF}^{-1} (5.4 ± 0.2 mmol·g^{-1}), which can be assigned to the Brønsted acidity from the HSO_4^- and μ_3-OH groups on the framework, as well as the adsorbed sulfate species in the pores. This value is in good agreement with the theoretical value of 9.5 mol·mol_{MOF}^{-1} obtained from structural and elemental analysis. It is worth noting that the framework in MFM-808-SO_4 exhibits high stability in humidity and water, which is one of the most important characteristics for proton transfer and photocatalytic H_2 evolution.

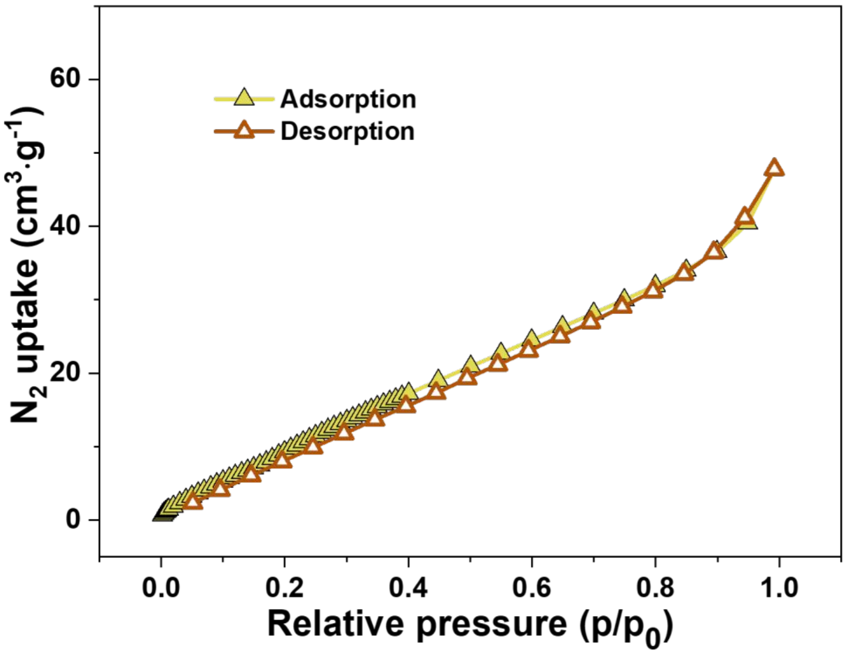

Figure S17. N_2 sorption isotherms of MFM-808-SO_4 at 77 K (BET: 47 $m^2.g^{-1}$).

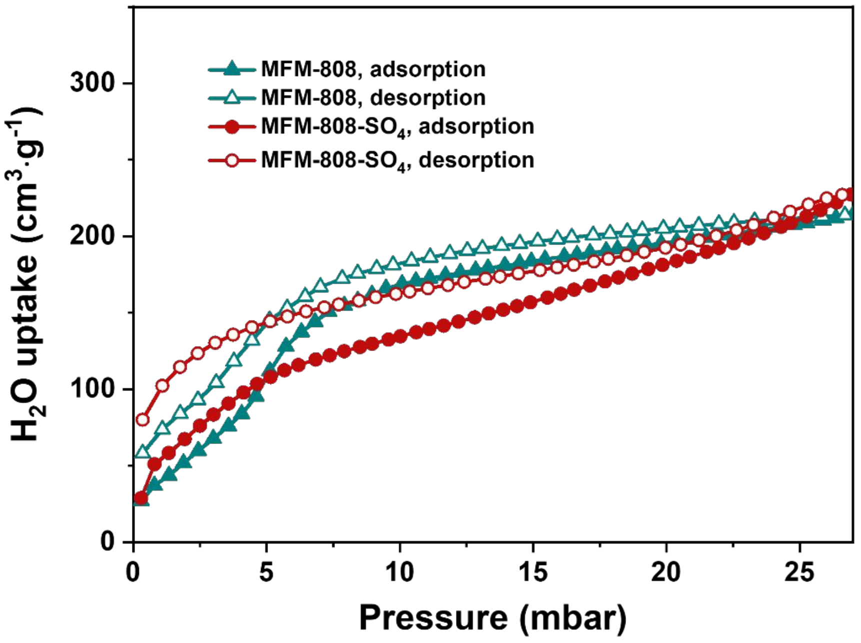

Figure S18. Water vapour sorption isotherms for MFM-808 and MFM-808-SO_4 at 298 K.

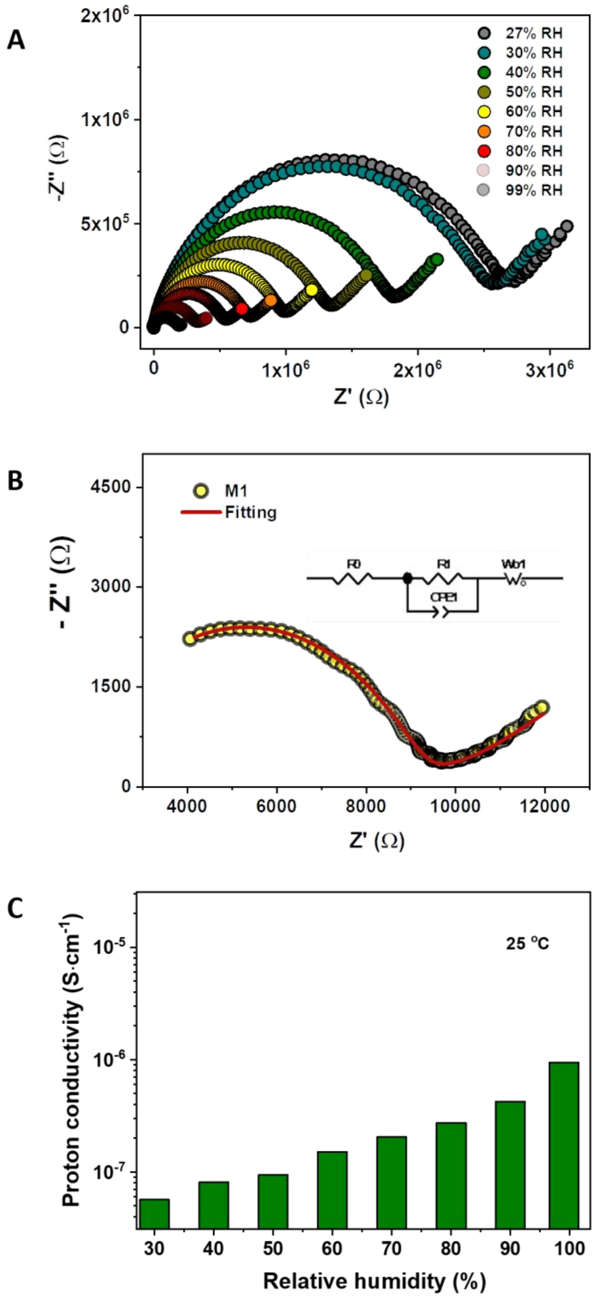

Figure S19. Impedance analysis for MFM-808 (RH dependence): (A) plot of the negative imaginary part (-Z’’) *vs* the real part (Z’) of measured impedance (Nyquist plots, frequency range: 1–10^6 Hz) at different RH (25 °C); (B) an example of using a proposed equivalent circuit for fitting the experimental data measured at 99% RH and 85 °C; (C) the obtained proton conductivity as a function of RH (25 °C).

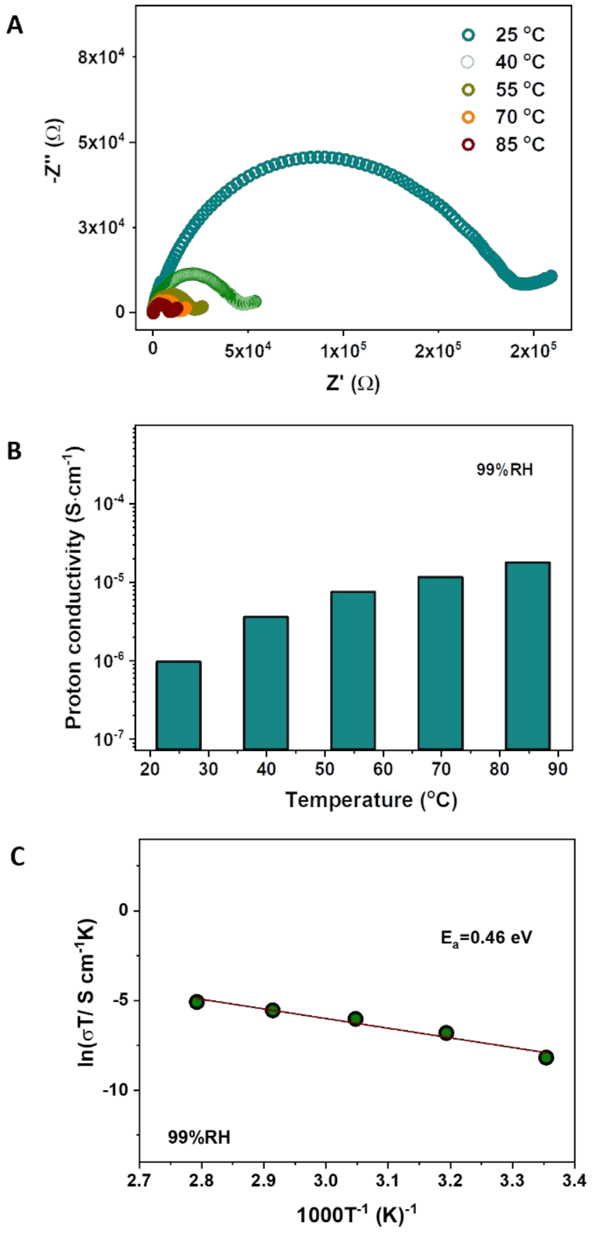

Figure S20. Impedance analysis for MFM-808 (temperature dependence): (A) Nyquist plots measured at various temperatures (99% RH); (B) proton conductivity as a function of temperature (99% RH); (C) the *Arrhenius* plot and derived activation energy.

The measured Nyquist plots (Figure S19A and S20A) can be fitted to the proposed equivalent circuits (Figure S19B). For the parent MFM-808, the derived value for σ showed a positive dependence on humidity (Figure S19C) and gave a value of 9.45×10^{-7} S.cm^{-1} under 99% RH (25 ºC). Increasing the temperature can improve the performance reaching 1.74×10^{-5} S.cm^{-1} at 85 ºC (99% RH, Figure S20B). This value is comparable with that of many pristine MOFs.[14] The corresponding activation energy (E_a) for MFM-808 was calculated to be 0.46 eV (Figure S20C).[15]

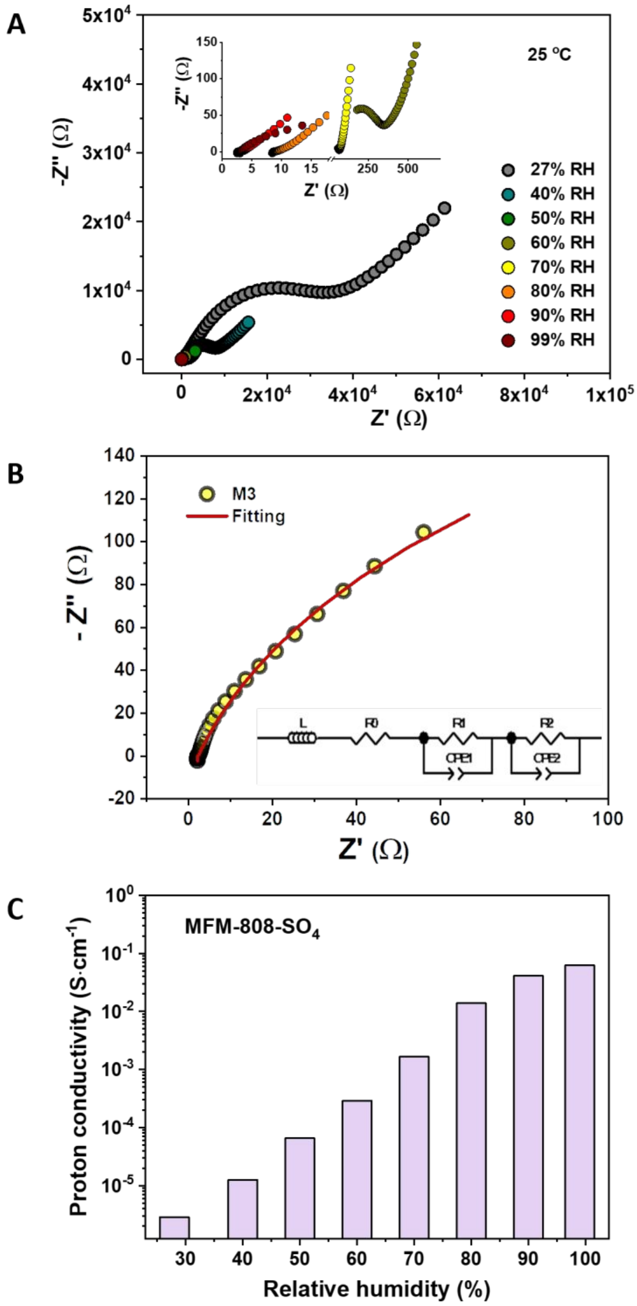

Figure S21. Impedance analysis for MFM-808-SO_4 (RH dependence): (A) Nyquist plots measured under various RH (25 °C). The inset is an enlargement of the high frequency region of the impedance complex plane. (B) an example of using a proposed equivalent circuit for fitting the experimental data measured at 99% RH and room temperature; (C) proton conductivity as a function of RH (25 °C).

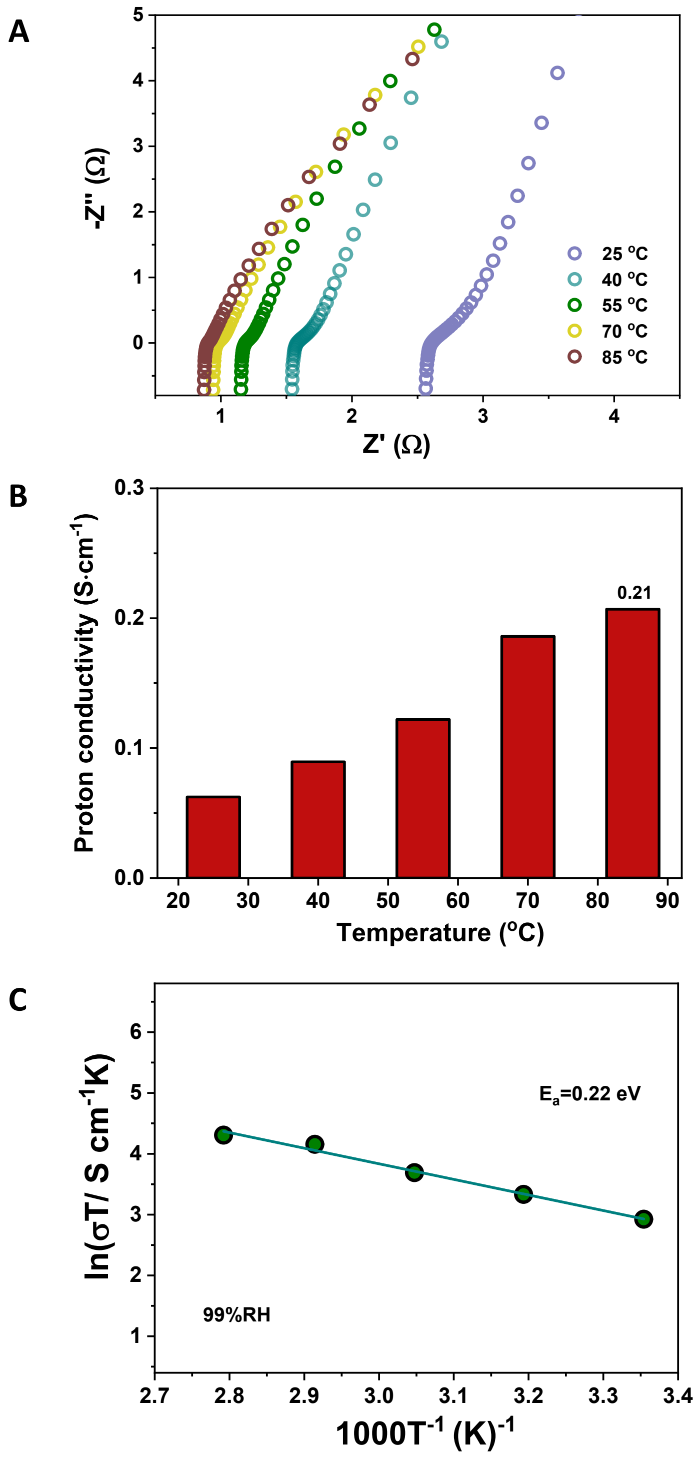

Figure S22. Impedance analysis for MFM-808-SO_4 (temperature dependence): (A) Nyquist plots measured at different temperatures (99% RH); (B) the obtained proton conductivity as a function of temperature (99% RH); (C) the *Arrhenius* plot and derived activation energy.

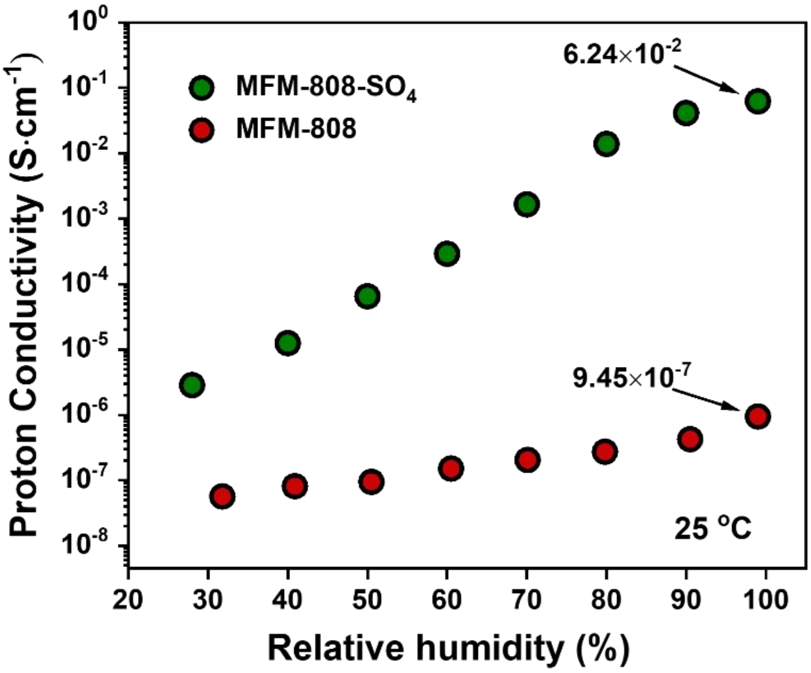

Figure S23. Proton conductivity measured for MFM-808 and MFM-808-SO_4 as a function of RH at 25 °C.

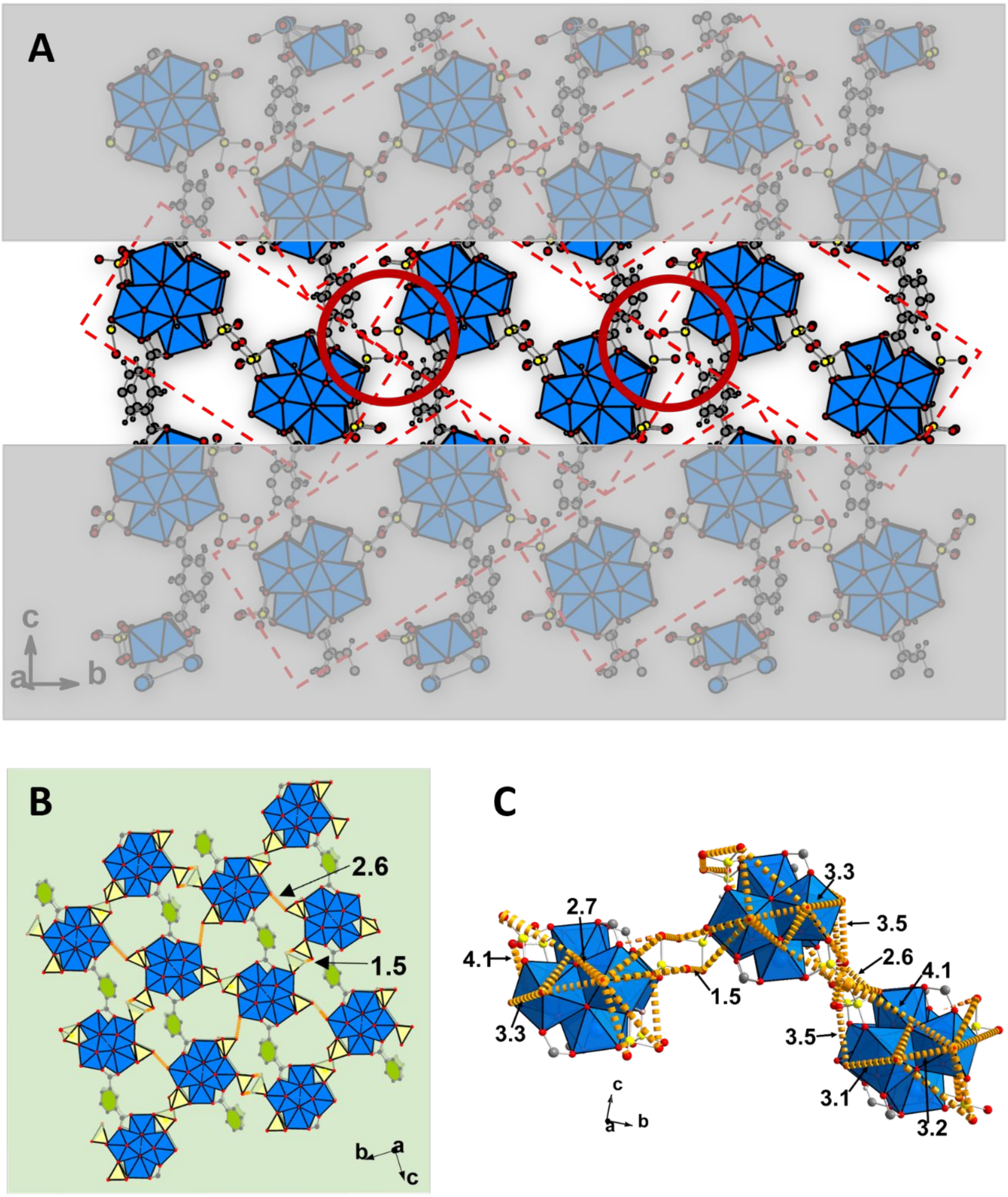

Figure S24. (A) View of adjacent $\{Zr_6\}$ clusters in the *bc* plane in MFM-808-SO_4. (B) View of the connection between $\{Zr_6\}_2$ pairs in the *bc* plane and (B) proton-transport pathways (orange dashed lines, distance in angstrom) between $\{Zr_6\}$ clusters in MFM-808-SO_4 (sulfate, yellow tetrahedral; aromatic rings, green; Zr_6, blue; O, red; S, yellow; C, grey).

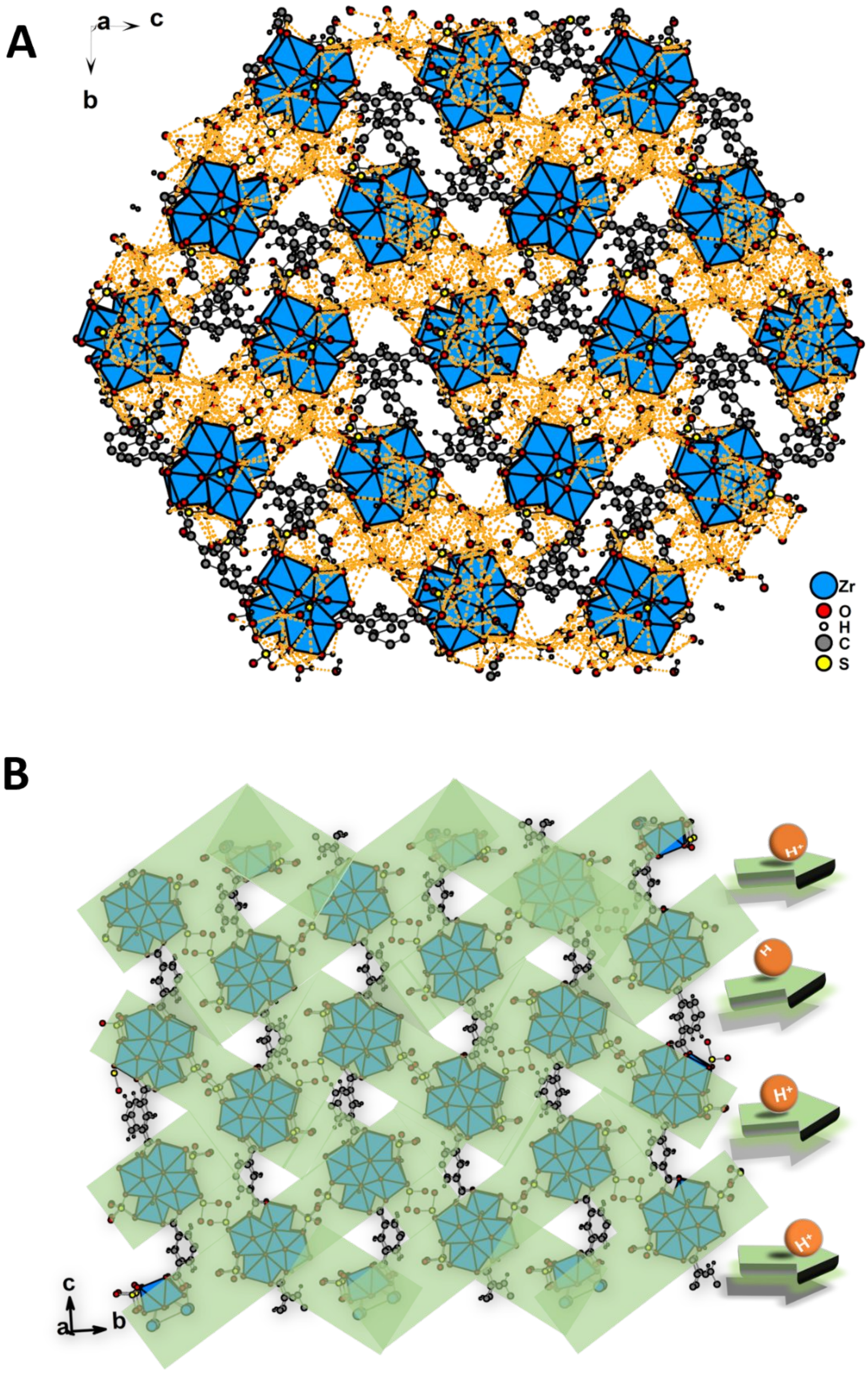

Figure S25. (A) View of potential hydrogen bonds (orange dashed lines; donor-acceptor distance ≤ 3.5 Å; Ĥ angle: 100–180°) within the layers of {Zr_6} clusters in MFM-808-SO_4. (B) Illustrations of potential routes/accessible areas (in green) for proton hopping on the {Zr_6}-layer in the *bc* plane in MFM-808-SO_4.

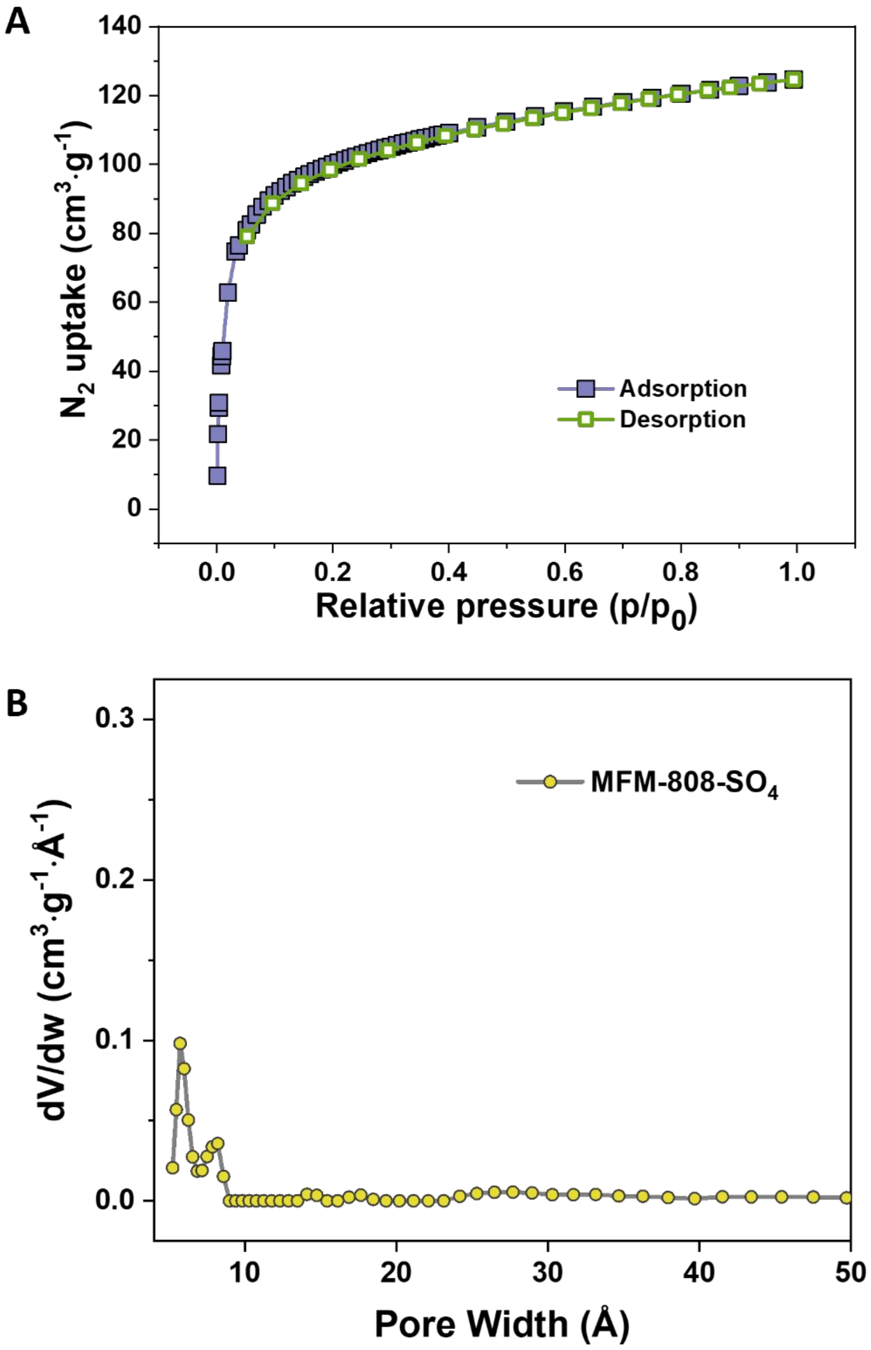

Figure S26. (A) N_2 sorption isotherms and (B) pore size distribution of MFM-808-SO_4-LL measured at 77 K (BET surface area = 326 $m^2.g^{-1}$).

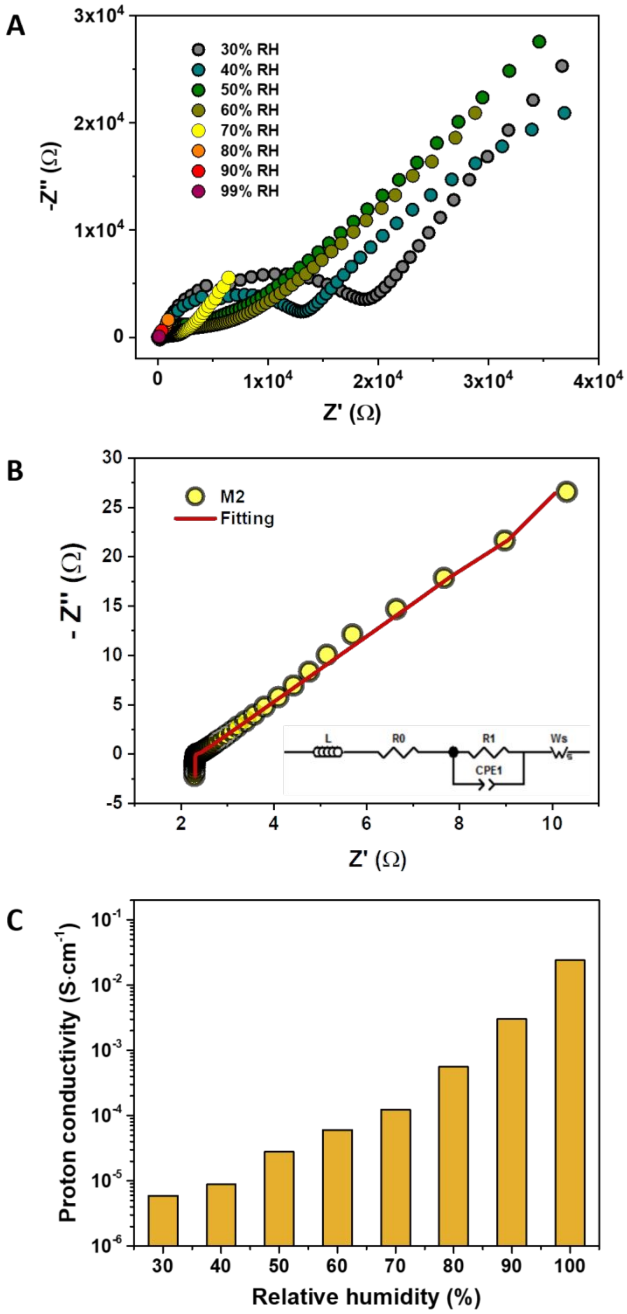

Figure S27. Impedance analysis for MFM-808-SO_4-LL (RH dependence): (A) Nyquist plots measured at different RH (25 °C); (B) an example of using a proposed equivalent circuit for fitting the experimental data measured at 99% RH and 85 °C; (C) proton conductivity as a function of RH (25 °C).

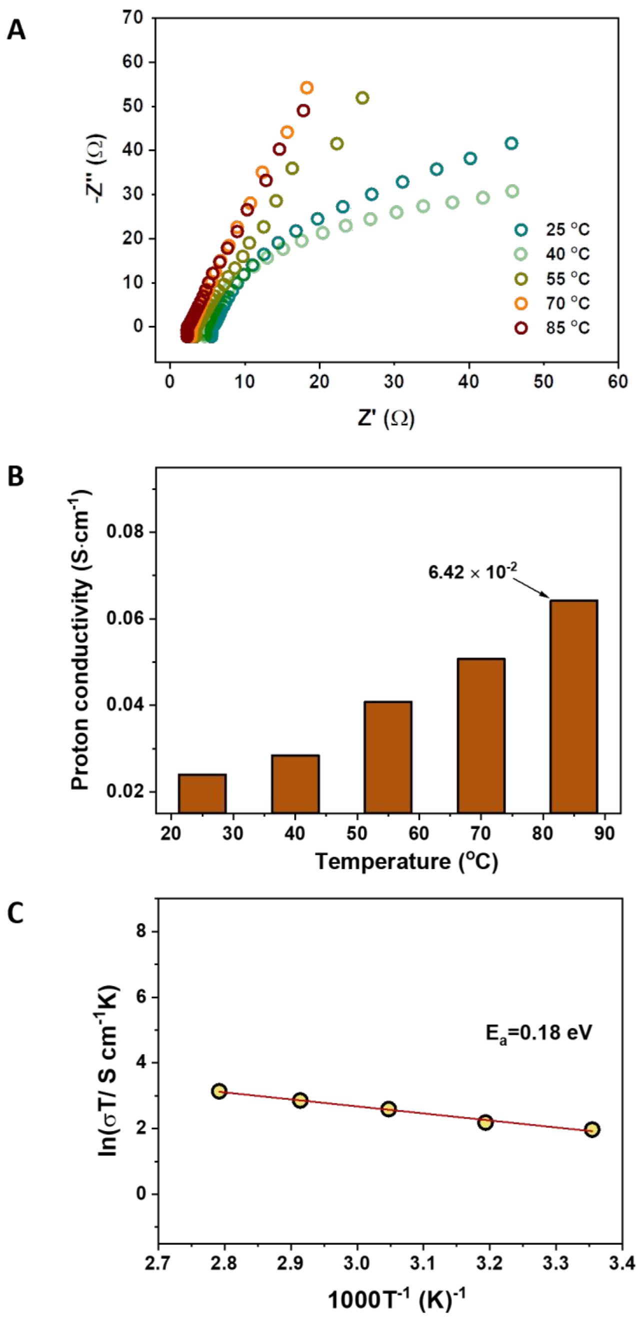

Figure S28. Impedance analysis for MFM-808-SO_4-LL (temperature dependence): (A) Nyquist plots measured at various temperatures (99% RH); (B) proton conductivity as a function of temperature (99% RH); (C) the *Arrhenius* plot and derived activation energy.

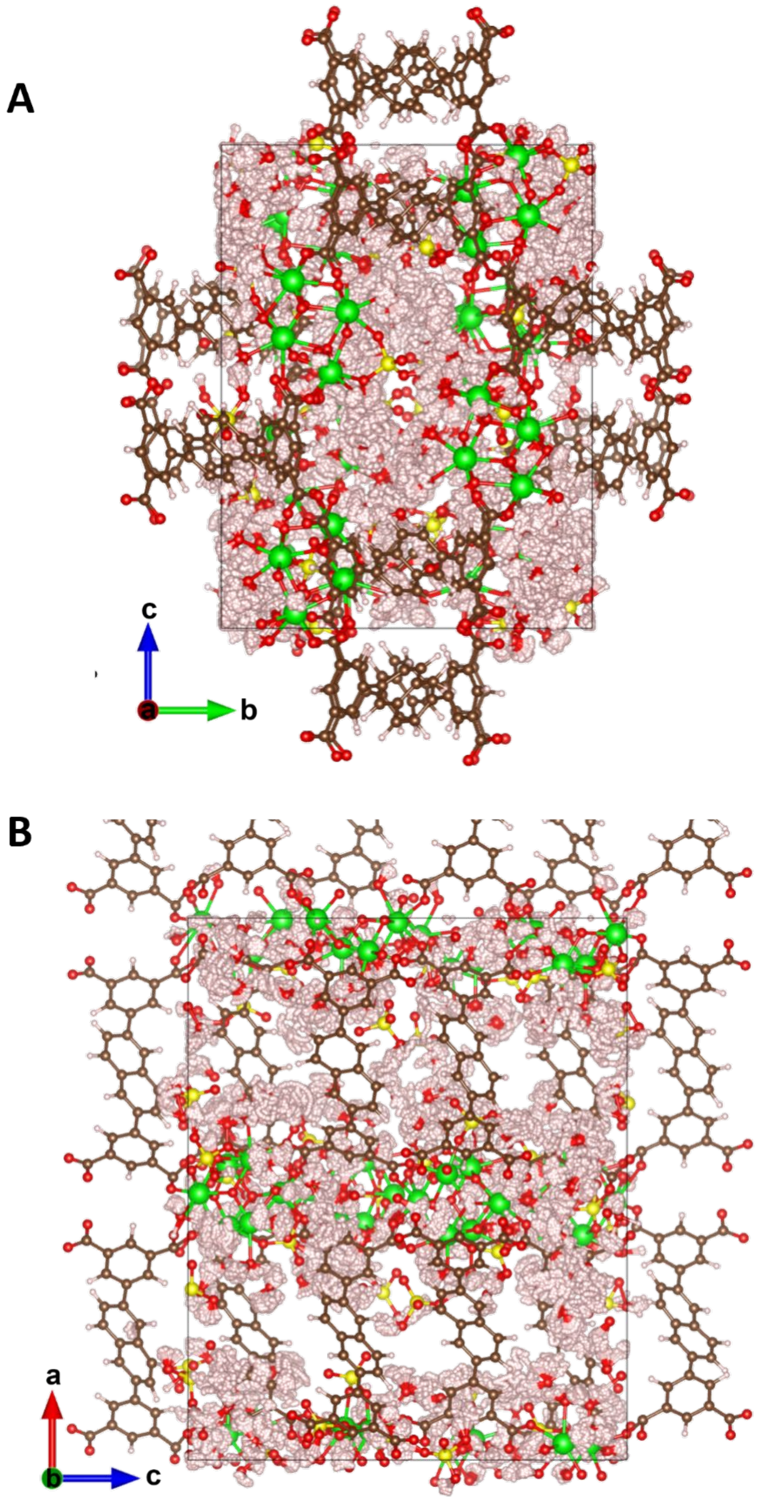

Figure S29. MD trajectories in MFM-808-SO_4 along (A) *a*-axis and (B) *b*-axis (H, mistyrose; O, red; C, brown; Zr, green; S, yellow).

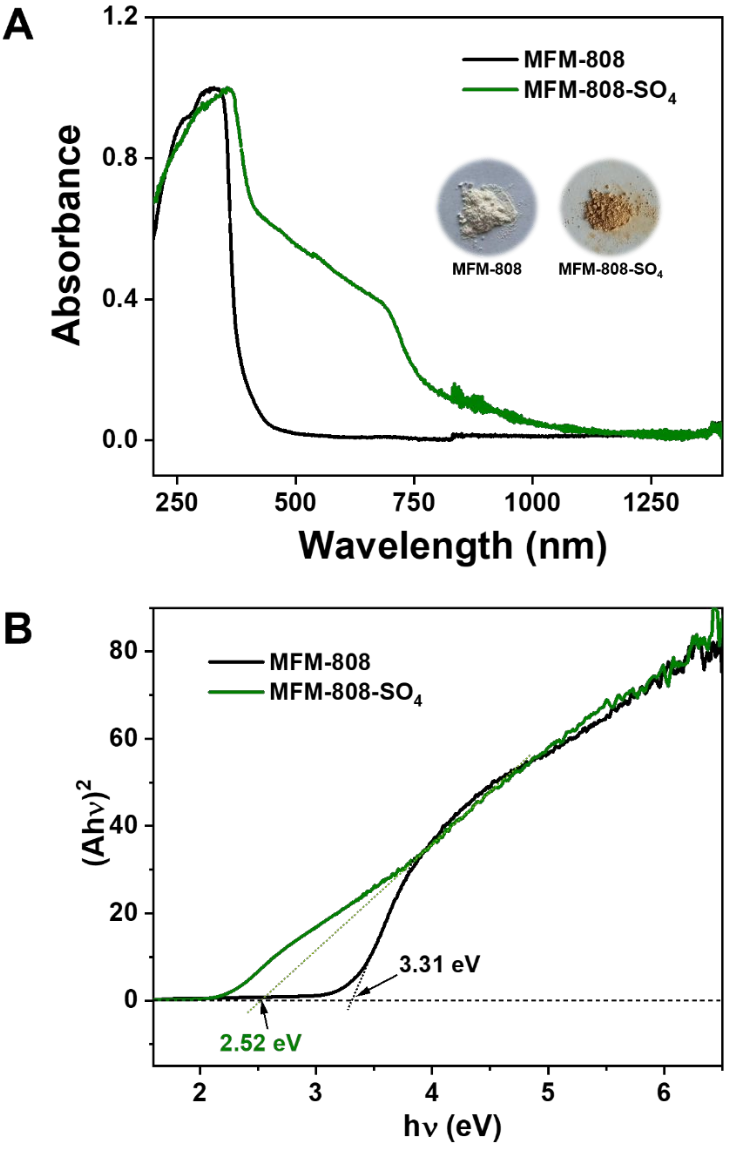

Figure S30. (A) UV-vis-DRS diffuse reflectance spectra and (B) the Tauc plots for MFM-808 and MFM-808-SO_4. The maximum band gap was calculated by replacing molar absorption coefficient (α) with absorbance (Abs) in the equation, $(\alpha h\nu)^2 = A(h\nu - E_g)$.

The optical absorption properties of MFM-808 and MFM-808-SO_4 were studied by UV-vis diffuse reflectance spectroscopy. Compared with MFM-808, the introduction of sulfate species in MFM-808-SO_4 contributes to the increased light absorption in the visible light region, which is responsible for the changes in color of the sample from white (MFM-808) to orange (MFM-808-SO_4). The visible light absorption spectrum of MFM-808-SO_4 thus differs significantly and is red-shifted with a high absorption intensity due to a *p*-π conjugation between the lone-pair electrons of the sulfate species (SO_4^{2-} and HSO_4^-) and the benzene and/or naphthalene rings of the organic ligands.[16] This highly conjugated system can donate electron density to the antibonding orbitals, which leads to a higher HOMO level that improves the optical absorption property. The HOMO-LUMO band gap energy calculated from Tauc plots follows MFM-808-SO_4 (2.52 eV) < MFM-808 (3.31 eV).

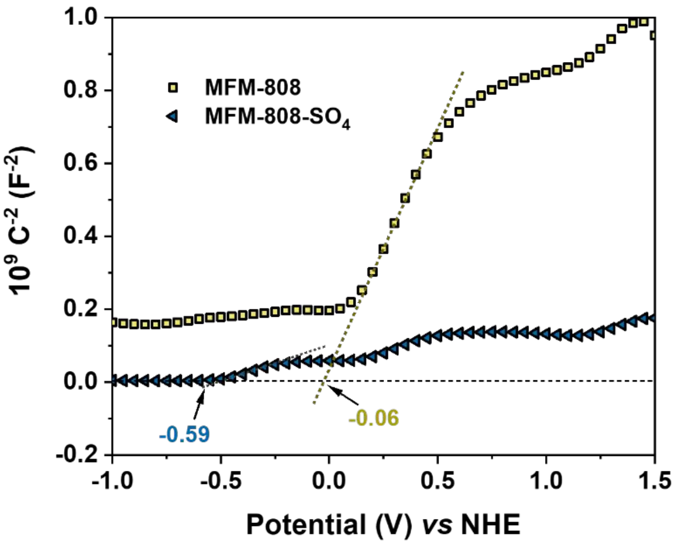

Figure S31. Mott–Schottky plots for MFM-808 and MFM-808-SO_4 at frequency of 1 kHz.

Mott–Schottky (MS) analysis was conducted to determine the position of conduction bands (CB) for MFM-808 and MFM-808-SO_4. The positive slope of the MS plots indicates that both MOFs are *n*-type semiconductors, and extrapolation of the MS plots yields a flat-band potential (*vs* NHE) of -0.59 V for MFM-808-SO_4 and -0.06 V for MFM-808, close to the value of the LUMO (E_{CB}). Together with the bandgaps, the position of valence bands (E_{VB} *vs* NHE) was calculated as +1.93 V for MFM-808-SO_4 and +3.25 V for MFM-808 (Figure 4D).

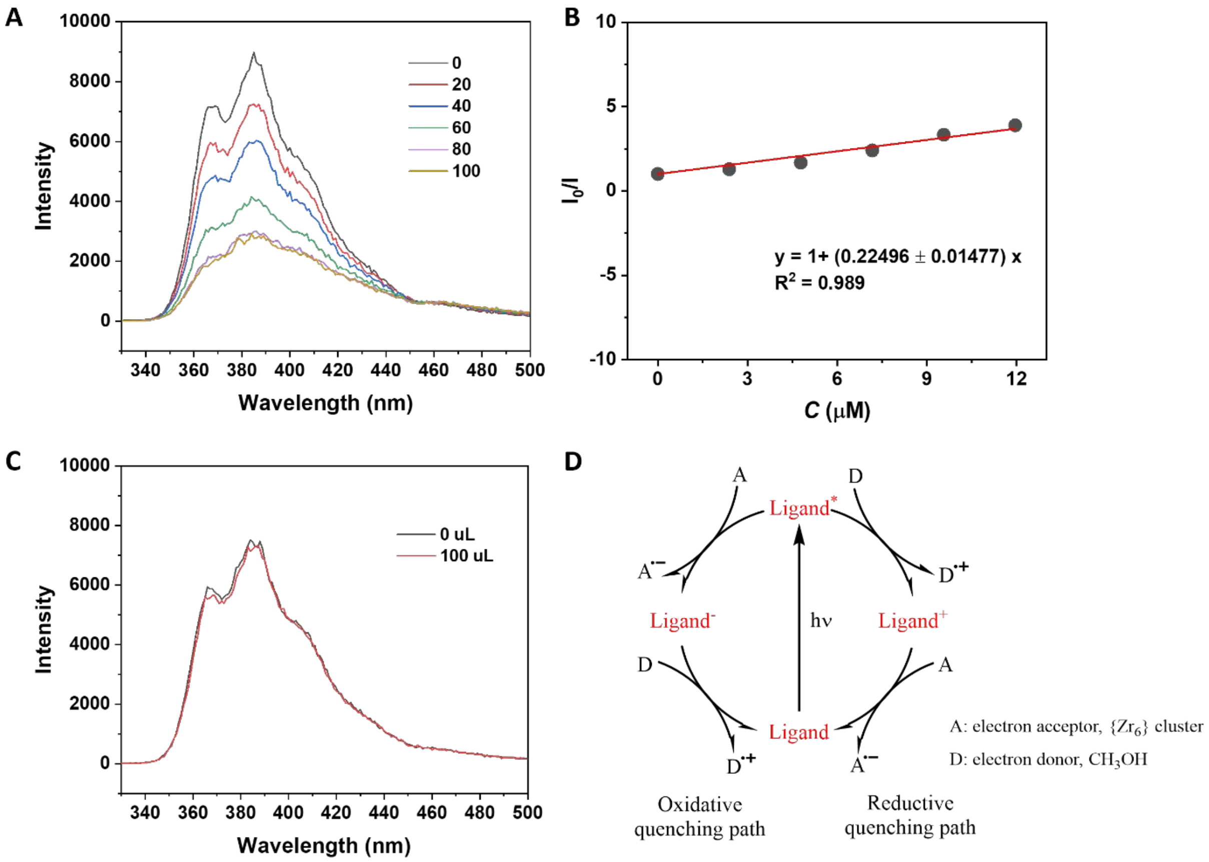

Figure S32. (A) Emission spectra of MFM-808-SO_4 after addition of different amounts of additional $\{Zr_6\}$ cluster using 320 nm excitation. (B) Plot of I_0/I (λ_{em} = 366 nm) for MFM-808-SO_4 as a function of the concentration of $\{Zr_6\}$ cluster (mM). (C) Emission spectra of MFM-808-SO_4 after the addition of CH_3OH using 320 nm excitation. (D) Proposed oxidative and reductive quenching path in photocatalysis. The $\{Zr_6\}$ cluster promotes an oxidative quenching pathway with electron transfer from the ligand/MOFs to the added cluster. Addition of CH_3OH promotes a reductive pathway *via* electron transfer from CH_3OH to ligand/MOFs. In this case, addition of $\{Zr_6\}$ cluster leads to decay, but not with addition of CH_3OH.

Photoluminescence and photocurrent responses were used to investigate the transfer of photo-excited electrons and recombination of electron-hole. The luminescence of MFM-808-SO_4 can be efficiently quenched by $\{Zr_6\}$ clusters (Figure S32A), but not quenched by the sacrificial agent, CH_3OH (Figure S32C). The luminescence quenching of MFM-808-SO_4 was fitted to the Stern–Völmer equation to afford a value for K_{sv} of 0.225 mM^{-1} (Figure S32B). These results suggest that the photocatalytic HER over MFM-808-SO_4 is initiated by an electron transfer from the photo-induced excited ligand L^* to the $\{Zr_6\}$ cluster. This was further supported by time-resolved photoluminescence measurements (Figure S33).

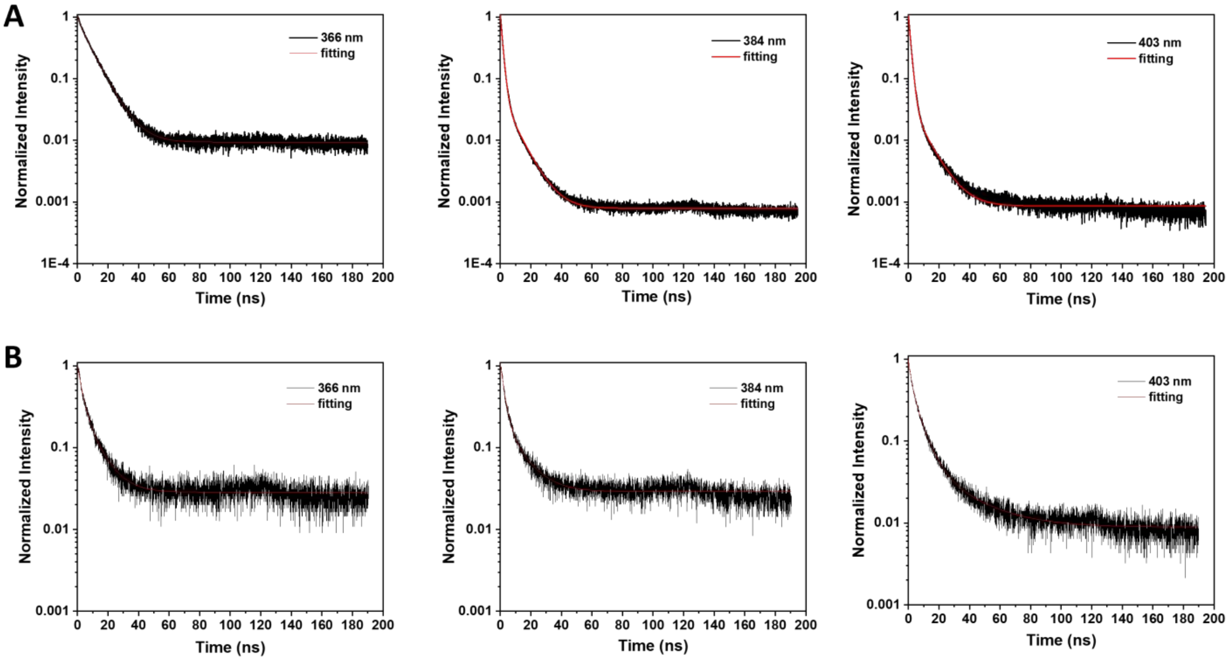

Figure S33. Normalized decay transients at 366 nm, 384 nm and 403 nm emission wavelength (using excitation at 375 nm) in an aqueous solution for (A) the ligand and (B) MFM-808-SO_4. The emission decays were fitted to the exponential expression $A = A_0 + A_1 e^{-t/\tau 1} + A_2 e^{-t/\tau 2}$ and to: $(\tau_{avg}) = \frac{A_1\tau_1^2 + A_2\tau_2^2}{A_1\tau_1 + A_2\tau_2}$.

The lifetimes of the excited state L^* in MFM-808-SO_4 were relatively short in comparison with that of the ligand and in physical mixtures of ligand and $\{Zr_6\}$ cluster (Table S6), indicating that the charge separation of photo-excited carriers is more efficient in MOFs. The electron transfers in MFM-808-SO_4 and in physical mixtures of ligand and $\{Zr_6\}$ cluster are both from the excited state L^* to $\{Zr_6\}$ clusters in terms of emission decay. The higher carrier separation efficiency of the MFM-808 materials was further confirmed by the photocurrent responses (Figure 4C). MFM-808-SO_4 exhibits a higher photocurrent density than that of MFM-808, which implies higher photo-absorbance and/or improved suppression of electron–hole recombination.

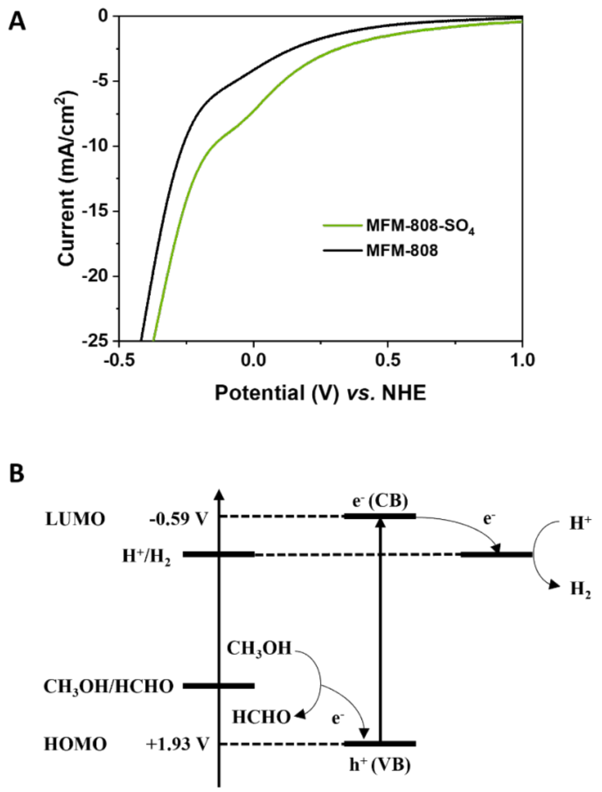

Figure S34. (A) Linear sweep voltammetry of MFM-808 and MFM-808-SO_4 coated on a glassy carbon electrode in 0.1 M Na_2SO_4 (aq, pH = 2.3) at a scan rate of 100 mV·s^{-1}. (B) Potential energy diagram of H_2 production from water over MFM-808-SO_4 as the photocatalyst with CH_3OH as an electron donor.

Electrochemical experiments were conducted to provide additional insights into the HER process. Since the pH value of MFM-808-SO_4 suspension solution in water is ~2.3, we used Na_2SO_4 (aq, pH = 2.3) as the electrolyte. Linear sweep voltammetry scans confirmed the HER catalytic peak of MFM-808-SO_4 with an onset potential of −0.152 V vs NHE (normalized hydrogen electrode) in a mixed solution with Na_2SO_4 (aq, pH = 2.3) and CH_3OH (V/V = 19/1). The linear sweep voltammetric curves and corresponding potential energy diagrams are shown in Figure S34. Compared to MFM-808, the smaller energy gap of MFM-808-SO_4 is consistent with its much higher HER activity (ΔE = 0.438 V, 670 μmol·g^{-1}·h^{-1}).

It is proposed that upon illumination, the photon antennae (*i.e.,* the organic ligands and sulfate species) are excited to produce photogenerated electrons and holes in MFM-808-SO_4. Photoelectrons then transfer to the {Zr_6} clusters in the layer, which plays an important role in preventing electron-hole recombination. In addition, potentiometric acid–base titrations quantified the concentration of Brønsted acid sites, and gave pK_{a1} = 2.60 ± 0.03 and pK_{a2} = 3.41 ± 0.06 derived from HSO_4^- and μ_3-OH groups in the framework (Figure S16). Thus, MFM-808-SO_4 accelerates the production of H^+ in water to form acidic solutions. Since the half reaction of water splitting to H_2 is:

$$H^+ + e^- \rightarrow \frac{1}{2}H_2$$

low pH can reduce the potential of this reaction. Proton transfer through the network can take place within the van der Waals bond distance, and such close contact can also facilitate electron transfer to the proton to generate H_2 and thus drives photocatalytic H_2 production from water.

4. Supplementary Tables

Table S1. Single crystal data and structural refinement for MFM-808 and MFM-808-SO_4.

	MFM-808	MFM-808-SO_4
Formula	$C_{34.5}$ $H_{33.5}$ N $O_{32.5}$ Zr_6	C_{26} $H_{51.5}$ $O_{55.7}$ $S_{5.5}$ Zr_6
Molar mass (g.mol^{-1})	1529.44	1978.54
Crystal system	Monoclinic	Monoclinic
Space group	*I* 2/a	*I* 2/a
a (Å)	31.1177(4)	30.9235(8)
b (Å)	18.8223(3)	19.2124(5)
c (Å)	25.1893(3)	25.0882(4)
α (°)	90	90
β (°)	93.9522(13)	92.455(2)
γ (°)	90	90
Cell volume (Å^3)	14718.46(3)	14891.6(6)
Z	8	8
Radiation	Cu Kα (λ = 1.54184 Å)	Cu Kα (λ = 1.54184 Å)
Density (calcd, g.cm^{-3})	1.380	1.765
Crystal size (mm^3)	0.2 × 0.1 × 0.1	0.2 × 0.1 × 0.1
μ(mm^{-1})	7.336	9.047
F(000)	5980.0	7847.0
Completeness	99.8% (to 2θ = 135.4°)	99.9% (to 2θ = 152.0°)
Temperature (K)	100.01 K	100.03 K
2θ range for data collection	5.49° ~ 144.228°	5.416° ~151.962°
Index ranges	-38 ≤ h ≤ 38,<br>-23 ≤ k ≤ 23,<br>-31 ≤ l ≤ 30	-38 ≤ h ≤ 38,<br>-23 ≤ k ≤ 23,<br>-31 ≤ l ≤ 30
Reflections collected	114145	82992
Independent reflections	14476<br>[R_{int} = 0.0911, R_{sigma} = 0.0546]	15217<br>[R_{int} = 0.0878, R_{sigma} = 0.0574]
Goodness-of-fit on F^2	1.075	1.022
Final R indexes [I>=2σ(I)]	R_1 = 0.0546, wR_2 = 0.1503	R_1 = 0.0750, wR_2 = 0.1974
Final R indexes [all data]	R_1 = 0.0671, wR_2 = 0.1568	R_1 = 0.1038, wR_2 = 0.2180
CCDC number	2176976	2176977

Table S2. Comparison of MOFs with a proton conductivity of >10^{-2} $S.cm^{-1}$.

Materials	σ ($S.cm^{-1}$)	T (°C), RH	Refs
MFM-808-SO_4	2.1×10^{-1}	85, 99%	This work
H_2SO_4@MIL-101-SO_3H (3M)	1.82	70, 90%	17
PSM-1	1.64×10^{-1}	80, 95%	18
Cr-MIL-88B-PSA	1.58×10^{-1}	100, 85%	19
IM-UiO-66-AS	1.54×10^{-1}	80, 98%	20
Co-tri	1.49×10^{-1}	80, 98%	21
H_2SO_4(1M)@MIL-101(Cr)-NH-$(CH_2)_3SO_3H$	1.3×10^{-1}	90, 95%	22
BUT-8(Cr)A	1.27×10^{-1}	80, 100%	23
PCMOF2$^1/_2$(Tz)	1.17×10^{-1}	85 90%	24
PCMOF2$^1/_2$(Pz)	1.11×10^{-1}	85, 90%	24
UiO-66-$(SO_3H)_2$	8.4×10^{-2}	80, 90%	25
TfOH@MIL-101	8×10^{-2}	15, 60%	26
MOF-808-4SA-150	7.89×10^{-2}	60, 95%	27
Nafion	7.8×10^{-2}	25, 100%	23
SSP@ZIF-8-10%	5.0×10^{-2}	75, 95%	28
BUT-8(Cr)	4.63×10^{-2}	80, 100%	23
$[(Me_2NH_2)_3(SO_4)]_2[Zn_2(ox)_3]$	4.2×10^{-2}	25, 98%	29
[Co(DCDPP)].$5H_2O$	3.9×10^{-2}	80, 97%	30
$[Gd_2(H_3nmp)_2].xH_2O$	3.79×10^{-2}	94, 98%	31
PCMOF10	3.55×10^{-2}	70, 95%	32
NiOF	3.41×10^{-2}	55, 95%	33
BUT-77	3.08×10^{-2}	80, 100%	34
MOF-74(Mg)-urea	2.64×10^{-2}	25, 95%	15
PCMOF2$^1/_2$	2.1×10^{-2}	85, 90%	35
KAUST-7’	2.0×10^{-2}	90, 95%	36
$[Pt(dach)(bpy)Br]_4(SO_4)_4$	1.7×10^{-2}	55, 95%	37
$[(CH_3)_2NH_2][In(TTFOC)]$	1.69×10^{-2}	70, 98%	38
Im-Fe-MOF	1.21×10^{-2}	60, 98%	39
PCC-72	1.2×10^{-2}	95, 95%	40
MIL-101-SO_3H	1.16×10^{-2}	80, 100%	23
MIP-202(Zr)	1.1×10^{-2}	90, 95%	41
H_2SO_4@MFM-555(Ho)	1×10^{-2}	20, 99%	42
H_3PO_4@MIL-101	1×10^{-2}	140, 1.1%	43
H_2SO_4@MIL-101	1×10^{-2}	150, 0.1%	43

Table S3. Calculated pK_a values for MFM-808-SO_4, UiO-66, UiO-67, NU-1000, and MOF-808[a].

MOF	pK_{a1}	pK_{a2}	pK_{a3}
	–HSO_4^-	μ_3-OH	–OH_2
MFM-808-SO_4	2.60 ± 0.03	3.41 ± 0.06	N.A.[b]
	pK_{a1}	pK_{a2}	pK_{a3}
MOF	μ_3-OH	–OH_2	–OH
Zr–UiO-67	3.44 ± 0.02	—	—
Zr–UiO-66	3.52 ± 0.02	6.79 ± 0.01	8.30 ± 0.02
Zr–UiO-67(HCl)	3.67 ± 0.01	6.6 ± 0.2	8.15 ± 0.02
NU-1000	3.59 ± 0.02	5.75 ± 0.04	8.2 ± 0.1
MOF-808	3.64 ± 0.01	6.22 ± 0.04	8.23 ± 0.04

[a]The p*K*a values of UiO series, NU-1000 and MOF-808 are from ref.[44]

[b]The equivalent point related to -OH_2 cannot be detected due to the decomposition of MOFs in alkali solution. The pK_a of Zr-OH is weak basic (pK_a > 8), which is not possible existence in MFM-808-SO_4.

Table S4. Comparison of the photocatalytic HER activity in MOFs (only pristine MOFs are included).

Catalysts	Irradiation	Sacrificial agents	solvents	H_2 evolution rate ($\mu mol.g^{-1}.h^{-1}$)	Refs
MFM-808-SO_4 (0.5 h)	>400 nm	CH_3OH	H_2O	1337	This work
MFM-808-SO_4 (24 h)	>400 nm	CH_3OH	H_2O	670	This work
ZrT-1-NH_2	> 400 nm	CH_3CN	H_2O	510	45
MIL-167	280 nm	TEA	CH_3CN	257	46
MIL-125-NH_2	>420 nm	TEOA	H_2O	167	47
NU-1000	> 400 nm	TEOA	H_2O	<50[a]	48
UiO-66	> 300 nm	TEOA	H_2O	29	49
UiO-66-NH_2	> 400 nm	CH_3CN	H_2O	25.1	50
	> 350 nm	CH_3OH	H_2O	11.8	
MOF-808	> 300 nm	TEOA	H_2O	7.8	51
MIL-100	>420 nm	CH_3OH	H_2O	5.9	52

[a]Estimated from the Figure in the reference.

Table S5. Comparison of the photocatalytic HER activity with Zr-based materials (including MOF-based composite, noble-metal-doped and decorated materials).

Catalysts	Irradiation	Sacrificial agents	solvents	H_2 evolution rate (μmol.g^{-1}.h^{-1})	Refs
MFM-808-SO_4 (0.5 h)	>400 nm	CH_3OH	H_2O	1337	This work
MFM-808-SO_4 (24 h)	>400 nm	CH_3OH	H_2O	670	This work
$Ni_3P_2W_{16}$@NU-1000	Full-optical	ascorbic acid	H_2O	13051	53
Zr-Bpydc-$PtCl_2$ MOCs	> 420 nm	TEOA	H_2O	10466	54
$Ni_3P_2W_{16}$@NU-1000	> 420 nm	ascorbic acid	H_2O	4948	53
$Ni_4P_2W_{18}$@UiO-68-Ir	> 400 nm	-	H_2O/MeOH	4400	55
P_2W_{18}@UiO-68-Ru	> 420 nm	-	DMF/CH_3CN	2716	56
CdS/UiO-66-NH_2	420 nm	Na_2S, Na_2SO_3	H_2O	2556	57
ZIS@P10 ($ZnIn_2S_4$@PCN-224)	> 420 nm	Na_2S, Na_2SO_3	H_2O	1400	58
MoS_2/UiO-66-NH_2/GO	> 420 nm	TEOA	H_2O	1069	59
Pt/KTa(Zr)O_3	> 300 nm	-	H2O	878	60
BN-2 (BiOI@NU-1000)	> 400 nm	TEOA	H_2O	610	61
UiO-66-30 (ErB/Pt-UiO-66)	> 420 nm	ascorbic acid	H_2O	460	62
NH_2-UiO-66(Zr)/B-CTF-1	> 420 nm	TEOA	H_2O	420	63
$BaZr_{0.7}Sn_{0.3}O_3$	Hg lamp	-	H_2O	280	51
Pd@MOF-808-b	> 300 nm	TEOA	H_2O	236	64
Pt@UiO-66-NH_2	Full-optical	MeOH	H_2O	220	49
ZrTTA-6SH-ZnTFPP	> 350 nm	TEOA	H_2O	110	65
UiO-66-Co_3O_4	> 300 nm	TEOA	H_2O	95	48
$Ce_{0.3}Zr_{0.7}O_{1.88}N_{0.12}$	> 300 nm	Na_2S, Na_2SO_3	H_2O	85	66
ZrTTA-6SH-NiTFPP	> 350 nm	TEOA	H_2O	71	65
ZrTTA-6SH-FeTFPP	> 350 nm	TEOA	H_2O	17	65
1 wt%Pt@UiO-66	> 400 nm	-	EtOH	3.9	67

Table S6. The lifetime of ligand, MFM-808-SO_4 and its homogeneous control samples in the aqueous solution.[a]

Samples	Lifetime (ns)		
	λ_{em} = 366 nm	λ_{em} = 384 nm	λ_{em} = 403 nm
Ligand	40.5	8.44	8.06
MFM-808-SO_4	7.13	7.27	8.21
MFM-808	7.73	4.20[b]	5.66
Zr_6 cluster, ligand	11.21	7.26	7.01

[a]The emission decays were fitted to the exponential expression $A = A_0 + A_1e^{-t/\tau 1} + A_2e^{-t/\tau 2}$, and the lifetime $(\tau_{avg}) = \frac{A_1\tau_1^2 + A_2\tau_2^2}{A_1\tau_1 + A_1\tau_1}$.

[b]The Instrument Response Function (IRF) was measured to be approximately 3ns FWHM in a 1% scattering solution of Ludox LS colloidal silica. The IRF was limited by the 5 ns bins in the MSA-300 card.

5. Supplementary References

(1) Lin, X.; Telepeni, I.; Blake, A. J.; Dailly, A.; Brown, C. M.; Simmons, J. M.; Zoppi, M.; Walker, G. S.; Thomas, K. M.; Mays, T. J.; Hubberstey, P.; Champness, N. R.; Schröder, M. High capacity hydrogen adsorption in Cu(II) tetracarboxylate framework materials: the role of pore size, ligand functionalization, and exposed metal sites. *J. Am. Chem. Soc.* **2009**, *131*, 2159.

(2) CrysAlis, C. In *CrysAlis Red*; Oxford Diffraction Ltd.: Abingdon, England, 2008.

(3) Dolomanov, O. V.; Bourhis, L. J.; Gildea, R. J.; Howard, J. A.; Puschmann, H. OLEX2: a complete structure solution, refinement and analysis program. *J. Appl. Cryst.* **2009**, *42*, 339.

(4) Hutter, J.; Iannuzzi, M.; Schiffmann, F.; VandeVondele, J. cp2k: atomistic simulations of condensed matter systems. *WIREs Comput. Mol. Sci.* **2014**, *4*, 15.

(5) Lippert, B. G.; Parrinello, J. H.; Michele A hybrid Gaussian and plane wave density functional scheme. *Mol. Phys.* **1997**, *92*, 477.

(6) VandeVondele, J.; Krack, M.; Mohamed, F.; Parrinello, M.; Chassaing, T.; Hutter, J. Quickstep: Fast and accurate density functional calculations using a mixed Gaussian and plane waves approach. *Comput. Phys. Commun.* **2005**, *167*, 103.

(7) VandeVondele, J.; Hutter, J. Gaussian basis sets for accurate calculations on molecular systems in gas and condensed phases. *J. Chem. Phys.* **2007**, *127*, 114105.

(8) Goedecker, S.; Teter, M.; Hutter, J. Separable dual-space Gaussian pseudopotentials. *Phys. Rev. B* **1996**, *54*, 1703.

(9) Perdew, J. P.; Burke, K.; Ernzerhof, M. Generalized gradient approximation made simple. *Physical Review Letters* **1996**, *77*, 3865.

(10) Grimme, S.; Antony, J.; Ehrlich, S.; Krieg, H. A consistent and accurate ab initio parametrization of density functional dispersion correction (DFT-D) for the 94 elements H-Pu. *J. Chem. Phys.* **2010**, *132*, 154104.

(11) Tauc, J.; Grigorovici, R.; Vancu, A. Optical properties and electronic structure of amorphous germanium. *Phys. Status Solidi B* **1966**, *15*, 627.

(12) Yuan, S.; Qin, J.-S.; Xu, H.-Q.; Su, J.; Rossi, D.; Chen, Y.; Zhang, L.; Lollar, C.; Wang, Q.; Jiang, H.-L.; Son, D. H.; Xu, H.; Huang, Z.; Zou, X.; Zhou, H.-C. [$Ti_8Zr_2O_{12}(COO)_{16}$] cluster: an ideal inorganic building unit for photoactive metal–organic frameworks. *ACS Cent. Sci.* **2018**, *4*, 105.

(13) Sarkisov, L.; Harrison, A. Computational structure characterisation tools in application to ordered and disordered porous materials. *Mol. Simul.* **2011**, *37*, 1248.

(14) Li, A.-L.; Gao, Q.; Xu, J.; Bu, X.-H. Proton-conductive metal-organic frameworks: Recent advances and perspectives. *Coord. Chem. Rev.* **2017**, *344*, 54.

(15) Sarango-Ramírez, M. K.; Lim, D.-W.; Kolokolov, D. I.; Khudozhitkov, A. E.; Stepanov, A. G.; Kitagawa, H. Superprotonic conductivity in metal–organic framework via solvent-free coordinative urea insertion. *J. Am. Chem. Soc.* **2020**, *142*, 6861.

(16) Nasalevich, M. A.; Becker, R.; Ramos-Fernandez, E. V.; Castellanos, S.; Veber, S. L.; Fedin, M. V.; Kapteijn, F.; Reek, J. N. H.; van der Vlugt, J. I.; Gascon, J. Co@NH_2-MIL-125(Ti): cobaloxime-derived metal–organic framework-based composite for light-driven H_2 production. *Energy Environ. Sci.* **2015**, *8*, 364.

(17) Li, X.-M.; Dong, L.-Z.; Li, S.-L.; Xu, G.; Liu, J.; Zhang, F.-M.; Lu, L.-S.; Lan, Y.-Q.

Synergistic conductivity effect in a proton sources-coupled metal–organic framework. *ACS Energy Lett.* **2017**, *2*, 2313.

(18) Mukhopadhyay, S.; Debgupta, J.; Singh, C.; Sarkar, R.; Basu, O.; Das, S. K. Designing UiO-66-based superprotonic conductor with the highest metal–organic framework based proton conductivity. *ACS Appl. Mater. Interfaces* **2019**, *11*, 13423.

(19) Liu, S.-S.; Han, Z.; Yang, J.-S.; Huang, S.-Z.; Dong, X.-Y.; Zang, S.-Q. Sulfonic groups lined along channels of metal–organic frameworks (MOFs) for super-proton conductor. *Inorg. Chem.* **2020**, *59*, 396.

(20) Li, X.-M.; Liu, J.; Zhao, C.; Zhou, J.-L.; Zhao, L.; Li, S.-L.; Lan, Y.-Q. Strategic hierarchical improvement of superprotonic conductivity in a stable metal–organic framework system. *J. Mater. Chem. A* **2019**, *7*, 25165.

(21) Elahi, S. M.; Chand, S.; Deng, W.-H.; Pal, A.; Das, M. C. Polycarboxylate-templated coordination polymers: role of templates for superprotonic conductivities of up to 10^{-1} S cm^{-1}. *Angew. Chem. Int. Ed.* **2018**, *57*, 6662.

(22) Devautour-Vinot, S.; Sanil, E. S.; Geneste, A.; Ortiz, V.; Yot, P. G.; Chang, J.-S.; Maurin, G. Guest-assisted proton conduction in the sulfonic mesoporous MIL-101 MOF. *Chem. Asian J.* **2019**, *14*, 3561.

(23) Yang, F.; Xu, G.; Dou, Y.; Wang, B.; Zhang, H.; Wu, H.; Zhou, W.; Li, J.-R.; Chen, B. A flexible metal–organic framework with a high density of sulfonic acid sites for proton conduction. *Nat. Energy* **2017**, *2*, 877.

(24) Kim, S.; Joarder, B.; Hurd, J. A.; Zhang, J.; Dawson, K. W.; Gelfand, B. S.; Wong, N. E.; Shimizu, G. K. H. Achieving superprotonic conduction in metal–organic frameworks through iterative design advances. *J. Am. Chem. Soc.* **2018**, *140*, 1077.

(25) Phang, W. J.; Jo, H.; Lee, W. R.; Song, J. H.; Yoo, K.; Kim, B.; Hong, C. S. Superprotonic conductivity of a UiO-66 framework functionalized with sulfonic acid groups by facile postsynthetic oxidation. *Angew. Chem. Int. Ed.* **2015**, *54*, 5142.

(26) Dybtsev, D. N.; Ponomareva, V. G.; Aliev, S. B.; Chupakhin, A. P.; Gallyamov, M. R.; Moroz, N. K.; Kolesov, B. A.; Kovalenko, K. A.; Shutova, E. S.; Fedin, V. P. High proton conductivity and spectroscopic investigations of metal–organic framework materials impregnated by strong acids. *ACS Appl. Mater. Interfaces* **2014**, *6*, 5161.

(27) Sharma, A.; Lim, J.; Jeong, S.; Won, S.; Seong, J.; Lee, S.; Kim, Y. S.; Baek, S. B.; Lah, M. S. Superprotonic conductivity of MOF-808 achieved by controlling the binding mode of grafted sulfamate. *Angew. Chem. Int. Ed.* **2021**, *60*, 14334.

(28) Liang, H.-Q.; Guo, Y.; Shi, Y.; Peng, X.; Liang, B.; Chen, B. A light-responsive metal–organic framework hybrid membrane with high on/off photoswitchable proton conductivity. *Angew. Chem. Int. Ed.* **2020**, *59*, 7732.

(29) Nagarkar, S. S.; Unni, S. M.; Sharma, A.; Kurungot, S.; Ghosh, S. K. Two-in-one: inherent anhydrous and water-assisted high proton conduction in a 3D metal–organic framework. *Angew. Chem. Int. Ed.* **2014**, *53*, 2638.

(30) Wu, H.; Yang, F.; Lv, X.-L.; Wang, B.; Zhang, Y.-Z.; Zhao, M.-J.; Li, J.-R. A stable porphyrinic metal–organic framework pore-functionalized by high-density carboxylic groups for proton conduction. *J. Mater. Chem. A* **2017**, *5*, 14525.

(31) Mendes, R. F.; Barbosa, P.; Domingues, E. M.; Silva, P.; Figueiredo, F.; Almeida Paz, F. A. Enhanced proton conductivity in a layered coordination polymer. *Chem. Sci.* **2020**, *11*,

6305.

(32) Ramaswamy, P.; Wong, N. E.; Gelfand, B. S.; Shimizu, G. K. H. A water stable magnesium MOF that conducts protons over 10^{-2} S·cm^{-1}. *J. Am. Chem. Soc.* **2015**, *137*, 7640.

(33) Gao, H.; He, Y.-B.; Hou, J.-J.; Zhang, X.-M. In situ aliovalent nickle substitution and acidic modification of nanowalls promoted proton conductivity in InOF with 1D helical channel. *ACS Appl. Mater. Interfaces* **2021**, *13*, 38289.

(34) Si, G.-R.; Yang, F.; He, T.; Kong, X.-J.; Wu, W.; Li, T.-C.; Wang, K.; Li, J.-R. Enhancing proton conductivity in Zr-MOFs through tuning metal cluster connectivity. *J. Mater. Chem. A* **2022**, *10*, 1236.

(35) Kim, S.; Dawson, K. W.; Gelfand, B. S.; Taylor, J. M.; Shimizu, G. K. H. Enhancing proton conduction in a metal–organic framework by isomorphous ligand replacement. *J. Am. Chem. Soc.* **2013**, *135*, 963.

(36) Mileo, P. G. M.; Adil, K.; Davis, L.; Cadiau, A.; Belmabkhout, Y.; Aggarwal, H.; Maurin, G.; Eddaoudi, M.; Devautour-Vinot, S. Achieving superprotonic conduction with a 2D fluorinated metal–organic framework. *J. Am. Chem. Soc.* **2018**, *140*, 13156.

(37) Otake, K.-i.; Otsubo, K.; Komatsu, T.; Dekura, S.; Taylor, J. M.; Ikeda, R.; Sugimoto, K.; Fujiwara, A.; Chou, C.-P.; Sakti, A. W.; Nishimura, Y.; Nakai, H.; Kitagawa, H. Confined water-mediated high proton conduction in hydrophobic channel of a synthetic nanotube. *Nat. Commun.* **2020**, *11*, 843.

(38) Su, J.; He, W.; Li, X.-M.; Sun, L.; Wang, H.-Y.; Lan, Y.-Q.; Ding, M.; Zuo, J.-L. High electrical conductivity in a 2D MOF with intrinsic superprotonic conduction and interfacial pseudo-capacitance. *Matter* **2020**, *2*, 711.

(39) Zhang, F.-M.; Dong, L.-Z.; Qin, J.-S.; Guan, W.; Liu, J.; Li, S.-L.; Lu, M.; Lan, Y.-Q.; Su, Z.-M.; Zhou, H.-C. Effect of imidazole arrangements on proton-conductivity in metal–organic frameworks. *J. Am. Chem. Soc.* **2017**, *139*, 6183.

(40) Qin, L.; Yu, Y.-Z.; Liao, P.-Q.; Xue, W.; Zheng, Z.; Chen, X.-M.; Zheng, Y.-Z. A "molecular water pipe": a giant tubular cluster {Dy_{72}} exhibits fast proton transport and slow magnetic relaxation. *Adv. Mater.* **2016**, *28*, 10772.

(41) Wang, S.; Wahiduzzaman, M.; Davis, L.; Tissot, A.; Shepard, W.; Marrot, J.; Martineau-Corcos, C.; Hamdane, D.; Maurin, G.; Devautour-Vinot, S.; Serre, C. A robust zirconium amino acid metal-organic framework for proton conduction. *Nat. Commun.* **2018**, *9*, 4937.

(42) Pili, S.; Rought, P.; Kolokolov, D. I.; Lin, L.; da Silva, I.; Cheng, Y.; Marsh, C.; Silverwood, I. P.; García Sakai, V.; Li, M.; Tang, C. C.; Yang, S.; Schröder, M. Enhancement of proton conductivity in nonporous metal–organic frameworks: the role of framework proton density and humidity. *Chem. Mater.* **2018**, *30*, 7593.

(43) Ponomareva, V. G.; Kovalenko, K. A.; Chupakhin, A. P.; Dybtsev, D. N.; Shutova, E. S.; Fedin, V. P. Imparting high proton conductivity to a metal–organic framework material by controlled acid impregnation. *J. Am. Chem. Soc.* **2012**, *134*, 15640.

(44) Klet, R. C.; Liu, Y.; Wang, T. C.; Hupp, J. T.; Farha, O. K. Evaluation of Brønsted acidity and proton topology in Zr- and Hf-based metal–organic frameworks using potentiometric acid–base titration. *J. Mater. Chem. A* **2016**, *4*, 1479.

(45) Sun, M.; Wang, Q.-Q.; Qin, C.; Sun, C.-Y.; Wang, X.-L.; Su, Z.-M. An amine-functionalized zirconium metal–organic polyhedron photocatalyst with high visible-light

activity for hydrogen production. *Chem. Eur. J.* **2019**, *25*, 2824.

(46) Assi, H.; Pardo Pérez, L. C.; Mouchaham, G.; Ragon, F.; Nasalevich, M.; Guillou, N.; Martineau, C.; Chevreau, H.; Kapteijn, F.; Gascon, J.; Fertey, P.; Elkaim, E.; Serre, C.; Devic, T. Investigating the case of titanium(IV) carboxyphenolate photoactive coordination polymers. *Inorg. Chem.* **2016**, *55*, 7192.

(47) Toyao, T.; Saito, M.; Horiuchi, Y.; Mochizuki, K.; Iwata, M.; Higashimura, H.; Matsuoka, M. Efficient hydrogen production and photocatalytic reduction of nitrobenzene over a visible-light-responsive metal–organic framework photocatalyst. *Catal. Sci. Technol.* **2013**, *3*, 2092.

(48) Li, X.; Gao, K.; Mo, B.; Tang, J.; Wu, J.; Hou, H. BiOI particles confined into metal–organic framework NU-1000 for valid photocatalytic hydrogen evolution under visible-light irradiation. *Inorg. Chem.* **2021**, *60*, 1352.

(49) Singh, A. K.; Gonuguntla, S.; Mahajan, B.; Pal, U. Noble metal-free integrated UiO-66-PANI-Co_3O_4 catalyst for visible-light-induced H_2 production. *Chem. Comm.* **2019**, *55*, 14494.

(50) Tian, P.; He, X.; Li, W.; Zhao, L.; Fang, W.; Chen, H.; Zhang, F.; Zhang, W.; Wang, W. Zr-MOFs based on Keggin-type polyoxometalates for photocatalytic hydrogen production. *J. Mater. Sci.* **2018**, *53*, 12016.

(51) Xu, J.; Liu, J.; Li, Z.; Wang, X.; Wang, Z. Synthesis, structure and properties of Pd@MOF-808. *J. Mater. Sci.* **2019**, *54*, 12911.

(52) Wang, D.; Song, Y.; Cai, J.; Wu, L.; Li, Z. Effective photo-reduction to deposit Pt nanoparticles on MIL-100(Fe) for visible-light-induced hydrogen evolution. *New J. Chem.* **2016**, *40*, 9170.

(53) Ji, C.; Wang, W.; El-Sayed, E.-S. M.; Liu, G.; Si, Y.; Su, K.; Ju, Z.; Wu, F.; Yuan, D. A high-efficiency dye-sensitized Pt(II) decorated metal-organic cage for visible-light-driven hydrogen production. *Appl. Catal. B: Environ.* **2021**, *285*, 119782.

(54) Jiao, L.; Dong, Y.; Xin, X.; Qin, L.; Lv, H. Facile integration of Ni-substituted polyoxometalate catalysts into mesoporous light-responsive metal-organic framework for effective photogeneration of hydrogen. *Appl. Catal. B: Environ.* **2021**, *291*, 120091.

(55) Kong, X.-J.; Lin, Z.; Zhang, Z.-M.; Zhang, T.; Lin, W. Hierarchical integration of photosensitizing metal–organic frameworks and nickel-containing polyoxometalates for efficient visible-light-driven hydrogen evolution. *Angew. Chem. Int. Ed.* **2016**, *55*, 6411.

(56) Zhang, Z.-M.; Zhang, T.; Wang, C.; Lin, Z.; Long, L.-S.; Lin, W. Photosensitizing metal–organic framework enabling visible-light-driven proton reduction by a wells–dawson-type polyoxometalate. *J. Am. Chem. Soc.* **2015**, *137*, 3197.

(57) Koganemaru, Y.; Kim, Y.; Watanabe, M.; Takagaki, A.; Ishihara, T. Z-scheme-type conductive-polymer-P3HT/KTa(Zr)O_3 heterojunction composites for enhancing the photocatalytic activity of water splitting. *Appl. Catal. A: Gen.* **2020**, *602*, 117737.

(58) Hao, X.; Jin, Z.; Yang, H.; Lu, G.; Bi, Y. Peculiar synergetic effect of MoS_2 quantum dots and graphene on metal-organic frameworks for photocatalytic hydrogen evolution. *Appl. Catal. B: Environ.* **2017**, *210*, 45.

(59) Jin, P.; Wang, L.; Ma, X.; Lian, R.; Huang, J.; She, H.; Zhang, M.; Wang, Q. Construction of hierarchical $ZnIn_2S_4$@PCN-224 heterojunction for boosting photocatalytic performance in hydrogen production and degradation of tetracycline hydrochloride. *Appl.*

Catal. B: Environ. **2021**, *284*, 119762.

(60) Li, F.; Wang, D.; Xing, Q.-J.; Zhou, G.; Liu, S.-S.; Li, Y.; Zheng, L.-L.; Ye, P.; Zou, J.-P. Design and syntheses of MOF/COF hybrid materials via postsynthetic covalent modification: an efficient strategy to boost the visible-light-driven photocatalytic performance. *Appl. Catal. B: Environ.* **2019**, *243*, 621.

(61) Chen, Y.-F.; Tan, L.-L.; Liu, J.-M.; Qin, S.; Xie, Z.-Q.; Huang, J.-F.; Xu, Y.-W.; Xiao, L.-M.; Su, C.-Y. Calix[4]arene based dye-sensitized Pt@UiO-66-NH_2 metal-organic framework for efficient visible-light photocatalytic hydrogen production. *Appl. Catal. B: Environ.* **2017**, *206*, 426.

(62) Wang, Y. L.; Jin, J. M.; Li, Y. H.; Wang, X. L.; Zhang, B.; Gong, X.; Wang, H. F.; Chen, A. P.; Zheng, L. R.; Hu, P.; Yang, H. G. $Ce_{0.3}Zr_{0.7}O_{1.88}N_{0.12}$ solid solution as a stable photocatalyst for visible light driven water splitting. *Appl. Catal. B: Environ.* **2018**, *224*, 733.

(63) Yuan, Y.-P.; Yin, L.-S.; Cao, S.-W.; Xu, G.-S.; Li, C.-H.; Xue, C. Improving photocatalytic hydrogen production of metal–organic framework UiO-66 octahedrons by dye-sensitization. *Appl. Catal. B: Environ.* **2015**, *168-169*, 572.

(64) He, J.; Wang, J.; Chen, Y.; Zhang, J.; Duan, D.; Wang, Y.; Yan, Z. A dye-sensitized Pt@UiO-66(Zr) metal-organic framework for visible-light photocatalytic hydrogen production. *Chem. Comm.* **2014**, *50*, 7063.

(65) Diao, Y.; Xu, N.; Li, M.-Q.; Zhu, X.; Xu, Z. Porphyrin grafting on a mercapto-equipped Zr(IV)-carboxylate framework enhances photocatalytic hydrogen production. *Inorg. Chem.* **2020**, *59*, 12643.

(66) Hou, W.; Chen, M.; Chen, C.; Wang, Y.; Xu, Y. Increased production of H_2 under visible light by packing CdS in a Ti, Zr-based metal organic framework. *J. Colloid Interface Sci.* **2021**, *604*, 310.

(67) Yuan, Y.; Zhao, Z.; Zheng, J.; Yang, M.; Qiu, L.; Li, Z.; Zou, Z. Polymerizable complex synthesis of $BaZr_{1-x}Sn_xO_3$ photocatalysts: role of Sn^{4+} in the band structure and their photocatalytic water splitting activities. *J. Mater. Chem.* **2010**, *20*, 6772.
